# Exercise and Prebiotic Fiber Provide Gut Microbiota-Driven Benefit in a Survivor to Germ-Free Mouse Translational Model of Breast Cancer

**DOI:** 10.3390/cancers14112722

**Published:** 2022-05-31

**Authors:** Kara Sampsell, Weilan Wang, Christina Ohland, Lukas F. Mager, Nicola Pett, Dana E. Lowry, Kate M. Sales, Margaret L. McNeely, Kathy D. McCoy, S. Nicole Culos-Reed, Raylene A. Reimer

**Affiliations:** 1 Faculty of Kinesiology, University of Calgary, Calgary, AB T2N 1N4, Canada; kara.sampsell@ucalgary.ca (K.S.); weilan.wang@ucalgary.ca (W.W.); dana.lowry@ucalgary.ca (D.E.L.); kmsales@ucalgary.ca (K.M.S.); nculosre@ucalgary.ca (S.N.C.-R.); 2 International Microbiome Centre, Cumming School of Medicine, University of Calgary, Calgary, AB T2N 4N1, Canada; christina.ohland@ucalgary.ca (C.O.); nicola.pett@ucalgary.ca (N.P.); 3 Department of Physiology and Pharmacology, Snyder Institute of Chronic Diseases, Cumming School of Medicine, University of Calgary, Calgary, AB T2N 4N1, Canada; lukas.mager@med.uni-tuebingen.de (L.F.M.); kathy.mccoy@ucalgary.ca (K.D.M.); 4 Department of Physical Therapy, University of Alberta, Edmonton, AB T6G 2R3, Canada; mmcneely@ualberta.ca; 5 Cancer Care Alberta, Department of Oncology, Edmonton, AB T5J 3E4, Canada; 6 Department of Oncology, Cumming School of Medicine, University of Calgary, Calgary, AB T2N 4N1, Canada; 7 Department of Biochemistry and Molecular Biology, Cumming School of Medicine, University of Calgary, Calgary, AB T2N 4N1, Canada

**Keywords:** gut microbiota, breast cancer, exercise, prebiotics, chemotherapy, fecal microbiota transplant

## Abstract

**Simple Summary:**

Breast cancer is the most common cancer in women worldwide. In recent years, the community of microbes that inhabit the intestinal tract, called the gut microbiota, has been shown to influence patient response to several cancer therapies. On the other hand, treatments such as chemotherapy can disrupt the resident gut microbiota and potentially contribute to poor health outcomes. Strategies to improve the composition of the gut microbiota include dietary and exercise interventions. While diet and exercise are already established as important for breast cancer prevention, during treatment, and for reducing recurrence, little is known about the impact of these factors on the gut microbiota in the context of breast cancer. Therefore, our aim was to examine the impact of exercise and diet on the gut microbiota in breast cancer. Our findings indicate that exercise and prebiotic fiber supplementation may provide benefits to individuals with breast cancer through advantageous gut microbial changes. Our findings of a potential adjuvant of exercise and prebiotics should inspire further mechanistic and clinical investigations.

**Abstract:**

The gut microbiota plays a role in shaping overall host health and response to several cancer treatments. Factors, such as diet, exercise, and chemotherapy, can alter the gut microbiota. In the present study, the Alberta Cancer Exercise (ACE) program was investigated as a strategy to favorably modify the gut microbiota of breast cancer survivors who had received chemotherapy. Subsequently, the ability of post-exercise gut microbiota, alone or with prebiotic fiber supplementation, to influence breast cancer outcomes was interrogated using fecal microbiota transplant (FMT) in germ-free mice. While cancer survivors experienced little gut microbial change following ACE, in the mice, tumor volume trended consistently lower over time in mice colonized with post-exercise compared to pre-exercise microbiota with significant differences on days 16 and 22. Beta diversity analysis revealed that EO771 breast tumor cell injection and Paclitaxel chemotherapy altered the gut microbial communities in mice. Enrichment of potentially protective microbes was found in post-exercise microbiota groups. Tumors of mice colonized with post-exercise microbiota exhibited more favorable cytokine profiles, including decreased vascular endothelial growth factor (VEGF) levels. Beneficial microbial and molecular outcomes were augmented with prebiotic supplementation. Exercise and prebiotic fiber demonstrated adjuvant action, potentially via an enhanced anti-tumor immune response modulated by advantageous gut microbial shifts.

## 1. Introduction

The community of bacteria, fungi, viruses, and protozoa inhabiting the human gastrointestinal tract, collectively known as the gut microbiota [1], can potentially facilitate or impede carcinogenesis and influence an individual’s response to certain cancer therapies [2,3]. This community of microbes acts alongside environmental exposures and epigenetic and genetic susceptibilities to shape cancer risk [3]. The mechanisms through which the gut microbiota exerts its influences on carcinogenesis and cancer treatments require further investigation. However, some relationships are understood to exist via microbiota-derived metabolites, modulation of host metabolism, and alterations to cytokine expression, the intestinal barrier, and immune regulation [4,5,6,7]. Harnessing the potential of the gut microbiota to improve treatment efficacy and health outcomes in cancer populations is of great clinical interest [4,8,9].

Current strategies for beneficially modifying the gut microbiota include improving dietary fiber intake [10], probiotic or prebiotic supplementation [11,12], and performing regular exercise [13,14]. Increasing the abundance of health-associated microbes and decreasing inflammation-associated microbes may improve markers of systemic inflammation, support gut barrier integrity, and decrease gastrointestinal side-effects and pathogenic infection risk [15]. Additionally, both prebiotic fiber and exercise have independently demonstrated positive immune and vascular modulatory effects on the tumor microenvironment, thus supporting the incorporation of these modifiable behaviors in cancer populations [16,17,18]. Both regular exercise and adequate fiber intake are currently recommended as protective lifestyle factors for individuals with cancer and for cancer prevention [19]. Additionally, individuals with breast cancer are at risk for disease recurrence and related mortality, which is positively associated with overweight and obesity [20,21] and negatively associated with regular exercise and adherence to nutritional recommendations [22,23]. These activities may also protect against altered inflammatory serum cytokines [24] and mitochondrial DNA mutations [25] that are implicated in breast cancer initiation and progression. However, research on applying strategies, such as exercise or prebiotic supplementation, with the aim of altering the gut microbiota in individuals with breast cancer is lacking.

In 2020, breast cancer comprised 11.7% of the 19.3 million new total cancer diagnoses and it accounts for one in four female cancer cases and one in six female cancer deaths [26]. Despite medical advances in detection, diagnostics, and treatment, which have improved survival, individuals with breast cancer comprise a large population which would benefit from improved treatment and health outcomes. Breast cancer is associated with an altered gut microbial profile compared to that of healthy controls [27,28,29], and treatments such as chemotherapy can further alter the gut microbiota toward dysbiosis [30,31,32,33]. Multiple studies have implicated the gut microbiota, and in some cases specific microbes, in successful response to various chemotherapies [8,9,34,35], suggesting similar mechanisms may support successful response to common breast cancer chemotherapeutics. Additionally, improved gut microbial profiles during or after treatment may help mitigate individuals’ increased risk for developing obesity or anxiety, depression, or fear of recurrence through favorable metabolic profile alterations [36,37] and the gut–brain axis [38,39,40,41,42] respectively. Up to 17% of those diagnosed with stage I and II breast cancer, 62% with stage III, and 66% with stage IV will undergo chemotherapy during their treatment [43]. Improving gut microbiota composition in this population is therefore a promising target to improve treatment and health outcomes and demands investigation.

The overall purpose of this study was to examine the effect of exercise and prebiotic supplementation on gut microbiota in a translational model of breast cancer. In the clinical portion of the study, the objective was to determine whether a 12-week exercise program alters gut microbiota composition in women with breast cancer who have undergone chemotherapy treatment. In the animal study, the objective was to use fecal microbiota transplant (FMT) to determine if colonization with human post-exercise gut microbiota would reduce tumor growth in germ free mice compared to pre-exercise gut microbiota, and whether prebiotic fiber could enhance the effect of post-exercise microbiota. All mice were injected with breast tumor cells and treated with the common chemotherapeutic Paclitaxel. Tight-junction protein gene expression and tumor and serum cytokine levels were analyzed as possible mechanistic links between the gut microbiota and tumor outcomes.

## 2. Materials and Methods

### 2.1. Clinical Study

#### 2.1.1. Alberta Cancer Exercise (ACE) Program

Study participants were recruited from individuals who had enrolled in the Alberta Cancer Exercise program (ACE). Participants adhered to the ACE study protocol which involved attending 12 weeks of bi-weekly 60-min exercise classes. Historically, this program was delivered in person but was shifted in March 2020 to a virtual delivery via Zoom due to the restrictions imposed by the COVID-19 pandemic. The exercise classes include aerobic, strength, and flexibility components as described in detail previously [44]. The intensity of the classes ranges from mild to moderate. Participants at any stage of cancer can be referred by a healthcare provider or directly contact ACE personnel to enroll in the program and may be actively in treatment or in survivorship up to 3 years post-treatment completion [44]. Several psychosocial and fitness measures are included in the ACE study protocol. For the purposes of this study, we accessed data on demographics, the Functional Assessment of Cancer Therapy-General (FACT-G) questionnaire, and Godin’s Leisure Time Exercise Questionnaire (GLTEQ), which are described below.

#### 2.1.2. Recruitment

Participants who met the inclusion criteria (Table 1) were sent an email by ACE personnel to inform them of the opportunity to participate. Following confirmation of eligibility, participants signed the informed consent document. Twenty-four individuals replied to the initial contact by ACE personnel, seventeen individuals expressed interest and were provided additional information and the eligibility questionnaire, four individuals were deemed ineligible, and three were no longer interested, leaving a cohort of ten participants.

#### 2.1.3. Sample Size and Power

**Figure 1:**
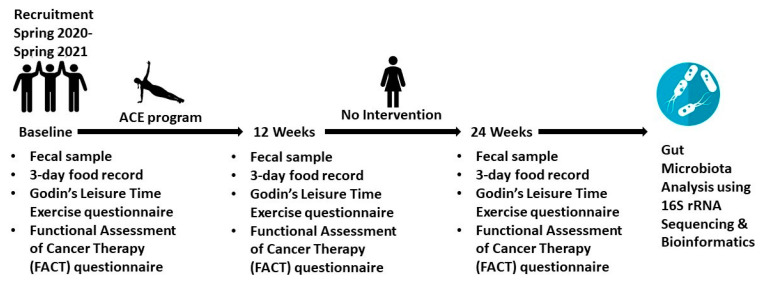
Clinical Study Outline. ACE, Alberta Cancer Exercise.

A target sample size of *n* = 26 was determined for this study. This target sample size was calculated based on previous studies on the gut microbiota of individuals with cancer or cancer survivors [45,46,47]. Additional considerations included a low expected drop-out rate based on historical compliance with the ACE program as well as the estimated size of the available target population. The given sample size would allow us to detect significant group differences (estimated with a power of 0.80, α = 0.05). Statistical calculations were performed utilizing an online statistical calculator provided by the University of British Columbia. Due to limitations resulting from the COVID-19 pandemic, our recruitment resulted in *n* = 10 participants recruited from four ACE sessions between Spring 2020 and Spring 2021. The outline of the study is provided below (Figure 1).

#### 2.1.4. Demographic Information

Demographic information related to age, ethnicity, education, income, and employment status as well as information on past treatment history, including whether they had received surgery, radiation, or hormone therapy in addition to chemotherapy, was collected.

#### 2.1.5. Godin’s Leisure Time Exercise Questionnaire (GLTEQ)

At baseline, 12 weeks (end of exercise program), and 24 weeks (12 weeks post-program), participants completed a GLTEQ as part of ACE. The questionnaire consists of four questions which query how frequently in a week the individual performs mild, moderate, or strenuous physical activity for a period of 15 min or more [48]. Time spent in mild, moderate, and strenuous exercise are multiplied by 3, 5, and 9, respectively, which are then totaled to yield a final score in metabolic equivalents (METs). This questionnaire has been found to correlate closely to measures of physical fitness such as VO_2_ max and is widely utilized in oncology research [49].

#### 2.1.6. Patient-Reported Psychosocial Outcomes

The Functional Assessment of Cancer Therapy (FACT-G) health-related quality of life questionnaire has 27 items and provides a cumulative score based on measures of physical, social, emotional, and functional well-being in cancer patients. The general questionnaire is designed and validated for use in any clinical cancer population [50]. Each item presents a statement to which the respondent is asked to choose a numeric rating from 0 to 4 to represent how the statement applies to them over the past 7 days. Total scores range from 0–108 with a higher numeric score indicating greater quality of life.

#### 2.1.7. Dietary Intake

Study participants documented their food intake in a 3-day dietary record (two weekdays and one weekend day) at 0, 12, and 24 weeks. Dietary records were analyzed using Food Works 18.0 software and the Canadian Nutrient File (The Nutrition Company, Long Valley, NJ, USA) [51].

#### 2.1.8. ACE Participant Fecal Samples and 16S rRNA Analysis

Participants collected stool samples using at-home collection kits and stored them in their home freezer until pick-up. Samples were picked-up and transported to the University of Calgary for storage at −80 °C within three days of collection. The gut microbial content was analyzed according to established protocols [52]. Bacterial DNA was extracted from ~250 mg of fecal sample using FastDNA Spin Kits (MP Biomedicals, Lachine, QC, Canada) with bead-beating and quantified using a PicoGreen DNA quantification kit (Invitrogen, Carlsbad, CA, USA). The V3–V4 regions of the 16S rRNA gene were amplified and sequenced on 2 × 300 bp MiSeq Illumina platform at the Centre for Health Genomics and Informatics (Calgary, AB, Canada) as previously described [53]. Demultiplexed 16S rRNA gene sequences were analyzed in QIIME2 platform using DADA2 for denoising and amplicon sequence variants (ASVs) extraction. ASV sequences were aligned to Silva 138 reference database and Genome Taxonomy Database for current taxonomy assignment. The resultant reads were analyzed using Shannon and Simpson indices, Weighted UniFrac, in QIIME2. Alpha and beta diversity analyses were calculated after rarefying the number of reads to 8683 for human samples and 10,011 reads for mouse samples using QIIME2 pipeline (version 2021.4) [54]. Differential abundance analysis was carried out with the unrarefied ASV counts table using the DESeq2 package in R (version 4.0.0).

### 2.2. Murine FMT Study

**Figure 2:**
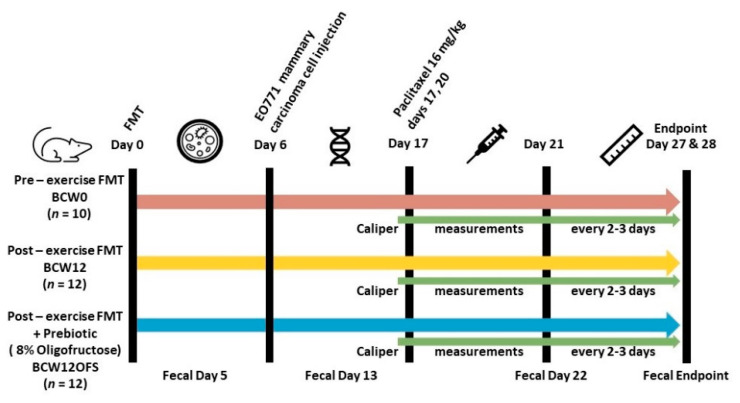
Murine Study Schematic.

A follow-up animal experiment was designed to investigate the relationship of exercise-responsive gut microbiota to breast cancer tumor growth and chemotherapy treatment. FMT allowed us to colonize germ-free mice with the pre- and post- exercise gut microbiota from a participant that demonstrated a favorable microbial response to exercise and assess tumor- and microbiota-related outcomes in the recipient mice. In addition to the exercise-responsive microbiota, supplementation with the prebiotic fiber oligofructose was also assessed in this murine breast cancer model to investigate a potential synergistic protective effect of exercise and prebiotic supplementation in breast cancer treatment. Based on the cost associated with running a study of this magnitude in the germ-free facility, one participant who showed a positive microbial effect to exercise was selected as the fecal donor for the FMT. An overview of the study design is provided in Figure 2.

#### 2.2.1. Animals

Forty-eight female 18–20-week-old C57BL/6 germ-free mice were bred and housed in the International Microbiome Centre (IMC) at the University of Calgary, Canada. All animals were kept on a 12-h light-dark cycle and fed standard chow. Animals were housed with litter mates in HEPA filtered iso-cages during the study. Animals were randomly allocated to four body weight and age-matched groups comprised of BCGF (germ-free control), BCW0 (received FMT of the participant’s baseline, pre-exercise fecal sample), BCW12 (received FMT of the participant’s 12-week, post-exercise fecal sample), and BCW12-OFS (received the same FMT as BCW12 and consumed oligofructose-supplemented water). Each group consisted of *n* = 12 mice at the start of the study. Two animals were euthanized in the BCW0 group following FMT, resulting in *n* = 10 mice in the BCW0 group.

#### 2.2.2. Cell Culture

The EO771 murine breast carcinoma cell line, originally isolated from a spontaneous tumor in a C57BL/6 mouse [55], was generously provided by the S. Liao lab at the University of Calgary. Cells were cultured in Dulbecco’s Modified Eagle Medium (DMEM, Gibco, ThermoFisher, Waltham, MA, USA) supplemented with 10% heat-inactivated fetal bovine serum (Gibco, ThermoFisher, Waltham, MA, USA). Cultures were maintained in an incubator at 37 °C and 5% CO_2_. Prior to injection, the cells were screened to ensure the absence of mycoplasma (PCR Mycoplasma detection kit, Thermo Scientific, Waltham, MA, USA).

#### 2.2.3. EO771 Cell Injections

To prepare injection aliquots, cells were detached from the flasks with trypsin. Once the cells were visibly detached, DMEM was added to inactivate the Trypsin. Cells were then pooled in a 50 mL Falcon tube, and an aliquot was removed to perform a cell count. The Falcon tube of cells was centrifuged for 5 min at 4 °C. Following centrifugation, the supernatant was removed, and the cells were resuspended in Dulbecco’s Phosphate Buffered Saline (DPBS; Sigma, Oakville, ON, Canada) to the proper concentration. The cell solution was aliquoted into designated microcentrifuge tubes for each group and diluted 1:1 with Corning^®^ Matrigel Matrix (Millipore Sigma, Oakville, ON, Canada) on ice to reach the final injection concentration. On day 6, each mouse received a 50 µL subcutaneous injection into the right flank which delivered 1 × 10^6^ EO771 cells.

#### 2.2.4. Paclitaxel Injections

On days 14 and 20, all mice were administered paclitaxel (Invitrogen, ThemoFisher, Waltham, MA, USA) dissolved in dimethyl sulfoxide (DMSO) and PBS via 100µL intraperitoneal injection. Paclitaxel is a common first-line chemotherapeutic for breast cancer which acts as a microtubule stabilizer, preventing mitotic cell division and inducing cell-death [56]. The mice received a cumulative 16 mg/kg dose over the two days in accordance with previously published work indicating dose tolerability [57]. The total dose was calculated based on the common low-dose used in breast cancer [58] with conversion from human to rodent dosing as per Reagan-Shaw et al. [59]. The solution was filtered with 0.2-micron filters to ensure sterility prior to injection.

#### 2.2.5. Oligofructose Supplementation

On day 0, the water bottles of the prebiotic group BCW12-OFS (*n* = 12) were replaced with oligofructose (Orafti P95, Beneo, Germany) solution which they consumed ad libitum to accrue an 8% dose of oligofructose for the remainder of the study. The oligofructose powder was weighed and mixed into water. The resultant solution was sterilized by filtration with a 0.2-micron filter to ensure sterility prior to consumption [60]. The dose calculation was based on an average 6 mL/mouse daily water intake and water bottles were weighed every third day to ensure adequate intake [61].

#### 2.2.6. Mouse Fecal Samples and 16S rRNA Analysis

Fecal samples were collected by handling the mice until they provided a sample directly into an autoclaved Eppendorf tube. A fecal sample was collected on day 5 to assess if the FMT had colonized the mice, on day 13 which was one week after the tumor cell injections, day 22 which was two days after the second paclitaxel injection, and on day 27 or 28 (endpoint). Fecal contents were stored at −80 °C. DNA extraction and sequencing was performed as described in Section 2.1.8. Analysis of the resultant reads was performed for Shannon and Simpson indices, Weighted UniFrac, and DESeq2 analysis as described above. Alpha and beta diversity analyses for the experimental groups were performed after rarefying the number of reads to 10,011 using QIIME2 pipeline (version 2021.4) [54]. Differential analysis was carried out with the unrarefied ASV counts table using the DESeq2 package in R (version 4.0.0) and controlled for cage effect in the model design.

#### 2.2.7. Tumor Measurements

Tumor measurements were taken every third day beginning on day 13 which is when tumors were consistently palpable (measurement days were 13, 16, 19, 22, 24, and 27 or 28 as endpoint). Tumor length and width were measured with metal calipers and the modified ellipsoid formula (V = 1/2(AB2)) was used to calculate subcutaneous tumor volume [62].

#### 2.2.8. Tissue Collection

Mice were euthanized on days 27 and 28. Half of the mice from each group were euthanized on each day due to the substantially increased time it takes to perform tasks in the germ-free facility. Mice were anesthetized with isoflurane and blood was collected via retro-orbital bleed. Blood was allowed to clot for 30 min, and serum was collected following a 10-min centrifugation at 4 °C and 2500 rpm. Cervical dislocation was performed followed by tumor resection and sampling of the distal ileum, proximal colon, and cecum. All tissues, fecal samples, and cecal contents were immediately snap-frozen in liquid nitrogen and stored at −80 °C until analysis.

#### 2.2.9. Tissue Real-Time PCR Analysis

Ileum and colon samples were processed using real-time PCR as previously described [63]. Total RNA was extracted using TRIzol reagent (Invitrogen, Carlsbad, CA, USA) and reverse transcription to cDNA performed using 2 µg of total RNA and cDNA synthesis kit (Invitrogen). Primers for ileal and colonic genes (zonula occludens (ZO-1), occludin, claudin-3) are listed in Table 2. The mRNA levels were calculated using the 2^−∆CT^ method [64].

#### 2.2.10. Serum and Tumor Cytokine Analysis

A panel of 31 cytokines, including Eotaxin, G-CSF, GM-CSF, IFNγ, IL-1α, IL-1β, IL-2, IL-3, IL-4, IL-5, IL-6, IL-7, IL-9, IL-10, IL-12 (p40), IL-12 (p70), IL-13, IL-15, IL-17A, IP-10, KC, LIF, LIX, MCP-1, M-CSF, MIG, MIP-1α, MIP-1β, MIP-2, RANTES, TNFα, and VEGF-A, were measured in serum and tumor homogenates by Eve Technologies (Calgary, AB, Canada) using BioPlex 200 Mouse Cytokine Array/Chemokine Array 31-Plex Milliplex Immunoassay (Millipore Sigma, Oakville, ON, Canada). The array of cytokines and chemokines in the panel is designed to analyze markers of immune activity, inflammation, and cancer.

#### 2.2.11. Statistical Analysis

Clinical Study: All data are presented as mean ± SEM. Data normality was tested using the Shapiro–Wilk normality test. GLTEQ, FACT-G, and dietary intake were analyzed using a paired samples *t*-test to compare time points. Significance is denoted as *p* < 0.05. Data analyses for non-microbial metrics were performed using SPSS statistics 27 (IBM). Further, 16S rRNA statistical analyses and Spearman’s correlations were completed in R (version 4.0.0). Alpha diversity was analyzed using a Kruskal–Wallis pairwise test in QIIME2 (version 2021.4). Beta diversity underwent analysis of similarity (ANOSIM) with 999 permutations. Adonis analysis using Weighted UniFrac values with 999 permutations was performed to investigate the %variance in beta diversity explained by exploratory factors.

Animal Study: All data are presented as mean ± SEM. Data normality was tested using the Shapiro–Wilk normality test. Tumor volume was analyzed using a repeated measures ANOVA (RMANOVA) with timepoint as the within-subject factor and group as the between-subject factor. Post-hoc Tukey tests were used to detect between group significance for all ANOVA analyses. One-way ANOVA was used to analyze single-timepoint data (e.g., tight junction proteins, tumor tissue cytokines, serum cytokines). Statistical analyses of non-microbial measures were performed with SPSS statistics 27 (IBM). Statistical analysis of 16S gut microbiota metrics was performed in R (version 4.0.0) as described above.

## 3. Results

### 3.1. Clinical Study Results: ACE’s Impact on Gut Microbiota in Breast Cancer Survivors

#### 3.1.1. Demographics

The demographics of the participants in the human ACE gut microbiota and breast cancer sub-study are presented in Table 3. Participants were primarily middle-aged (57.9 ± 2.8 years old), of European descent, and of high socioeconomic status. Occasional drinking was reported most frequently (50%) for alcohol consumption and 60% reported never smoking.

#### 3.1.2. Participant Clinical Characteristics

Table 4 provides the clinical characteristics of the participants. All participants in the study completed chemotherapy prior to the study start and had also undergone surgery for their breast cancer with 80% also having received radiation therapy. Treatments that coincided with the study period included hormone therapy for 50% of participants and zoledronic acid infusions for one participant. A total of 70% of participants were overweight (40%) or obese (30%), and one participant was underweight.

#### 3.1.3. Godin’s Leisure Time Exercise Questionnaire

The results of the GLTEQ are reported in Table 5. A significant difference in mean reported MET hours spent per week exercising was found between weeks 0 and 12 (*p* = 0.002) and weeks 0 and 24 (*p* = 0.030), but not between weeks 12 and 24 (*p* = 0.535). The increase from week 0 to week 12 coincides with the duration of the ACE program. Reported strenuous, moderate, and mild exercise increased from 0 to 12 weeks. However, only the increase in strenuous exercise was statistically significant (*p* = 0.016).

#### 3.1.4. Three-Day Food Record Dietary Analysis

Results of the three-day food record analysis for key nutrient intakes are provided in Table 6. There were no differences in macronutrient intake between 0 and 12 weeks. However, total caloric intake decreased from 12 to 24 weeks, dropping from 2260.2 ± 115.0 kcal/day to 1785.3 ±196.9 kcal/day (*p* = 0.017). During the post-ACE exercise (washout) period, total fat intake decreased from 107.4 ± 9.0 g/day to 74.2 ± 12.9 g/day (*p* = 0.012) between weeks 12 and 24 and included significant decreases in polyunsaturated and monounsaturated fat. Vitamin E intake and selenium intake also decreased significantly between weeks 12 and 24. No differences were found for cholesterol, calcium, copper, phosphorus, zinc, iron, magnesium, potassium, sodium, vitamin A, beta-carotene, B vitamins, vitamin C, vitamin D, Vitamin K, isoleucine, leucine, valine, butyric, alcohol, caffeine, and phytosterols (data not shown).

#### 3.1.5. Health-Related Quality of Life Results: FACT-G

The results of the Functional Assessment of Cancer Therapy–General questionnaire for each time point are summarized in Table 7. There were no significant changes in the total score or the four categories of well-being between baseline, week 12, and week 24.

#### 3.1.6. Gut Microbial Composition Suggests Some Response to Exercise

**Figure 3:**
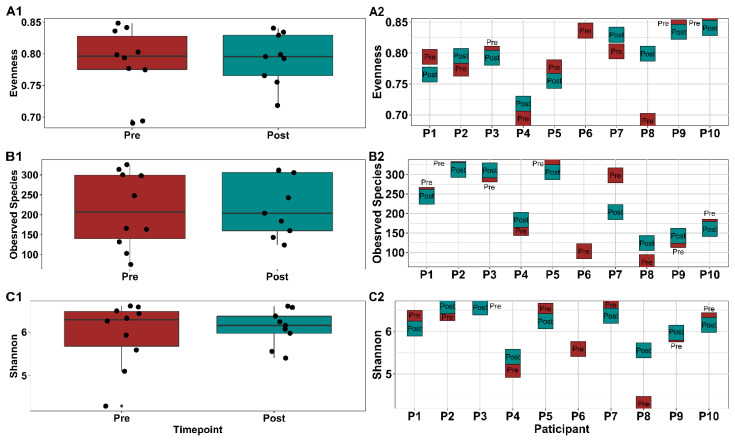
Gut microbiota alpha diversity indices in Alberta Cancer Exercise (ACE) participants. Measures of alpha diversity pre (baseline) and post (12 weeks) ACE program. (**A1**–**C1**) show pooled samples while (**A2**–**C2**) visualize pre and post samples for each participant individually. Metrics include Pielou’s Evenness (**A1**,**A2**), Observed Species (**B1**,**B2**), and Shannon (**C1**,**C2**). No statistical significance was found using Kruskal-Wallis pairwise tests.

Alpha diversity, a measure of microbial diversity within a sample, is shown in Figure 3. Alpha diversity indices weight two components, richness (count of the number of different taxa in the sample) and evenness (equitability of taxa frequencies in a sample). Gut microbial evenness, shown as pooled participant data (Figure 3(A1); *p* = 0.87) or assessed as individual data (Figure 3(A2)), did not differ between baseline (pre-exercise) and 12 weeks (post-exercise), nor between 12 weeks and the end of the washout period at 24 weeks (*p* = 0.15). Similarly, observed species, which measures richness, did not differ between pre- and post-samples (Figure 3(B1,B2)), nor between 12 and 24 weeks. The Shannon index, which equally weights evenness and richness, did not change over time (Figure 3(C1,C2)).

**Figure 4:**
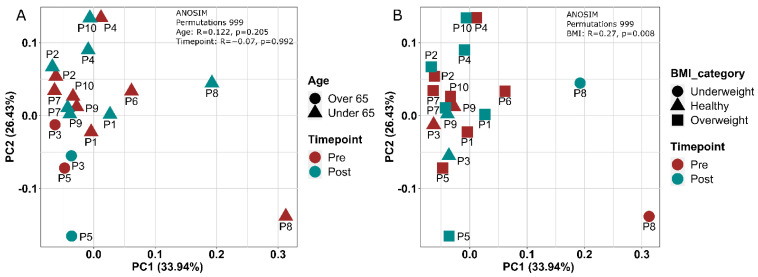
Gut microbiota beta diversity analyses for Alberta Cancer Exercise (ACE) participants. Beta diversity was measured by Weighted UniFrac Distance Matrix-based PCoA for pre- (baseline) and post- (12 weeks) exercise in the ACE program and analyzed with ANOSIM. Age (over or under 65) and time point did not significantly influence community diversity (**A**). Body mass index (BMI) category significantly influenced community diversity (*p* = 0.008) (**B**).

Analysis of beta diversity using weighted UniFrac distances indicated that gut microbial communities did not differ between baseline and 12 weeks (Figure 4A). An R value far below 1 (R = 0.122) indicated low dissimilarity between the communities. Had exercise significantly altered the microbial community structure, we would have expected to see distinct clustering of samples according to pre- and post-exercise time points. This was not the case and samples largely clustered together. Additional analysis was conducted to take age (over or under 65 years old) and BMI (underweight, healthy, overweight) into account as potential influencers of beta diversity. There was no difference in community structure based on age category (Figure 4A; *p* = 0.205), but there was a difference found when participant BMI was taken into account (Figure 4B). There was a significant (*p* = 0.008) dissimilarity in gut microbial structure (R = 0.27) according to BMI (Figure 4B), which was likely driven by the underweight participant. Overweight and obese BMI category groups were combined in this analysis due to the similar effects of these characteristics on gut microbial communities.

**Figure 5:**
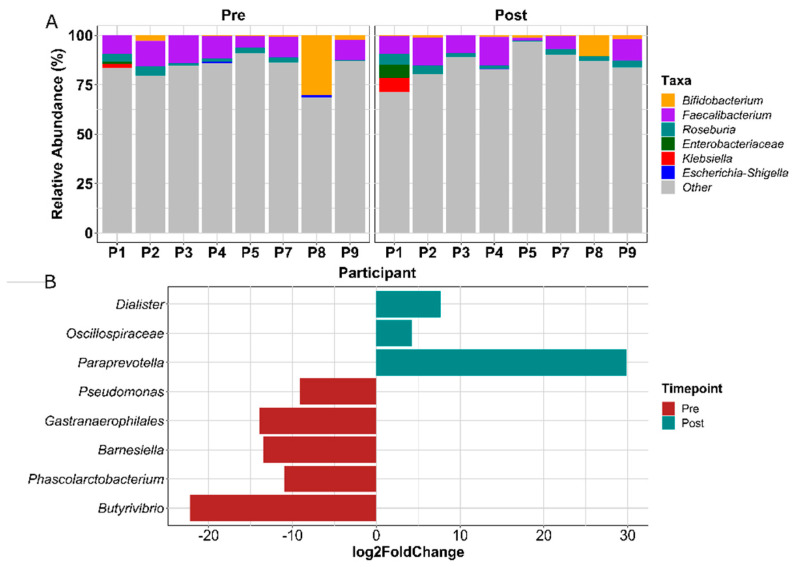
Gut microbiota differential abundance analyses from 16S rRNA sequencing. Relative abundance of three health-associated and three inflammation-associated microbiota were analyzed using DESeq2, showing no significant differences (**A**). Eight microbiota were significantly differentially abundant between pre- (baseline) and post- ACE (12 weeks) samples when analyzed with DESeq2 (*p* < 0.01) (**B**).

The relative abundance of three health-associated genera (*Bifidobacterium*, *Faecalibacterium*, *Roseburia*) [65,66,67,68] and three inflammation-associated genera (*Enterobacteriaceae*, *Klebsiella*, *Escherichia-Shigella*) [69,70,71] at pre-exercise and post-exercise time points are presented in Figure 5A. No significant differences were found in the relative abundance of these bacteria between time points (Figure 5A). Figure 5B presents the results of DESeq2 analysis to investigate whether any microbiota differed significantly between pre- and post-exercise time points with participants accounted for as a covariate in the analysis. The relative abundance of *Dialister*, *Oscillospiraceae*, and *Paraprevotella* was significantly higher in post-exercise samples compared to pre-exercise samples (*p* < 0.01) (Figure 5B). Pre-exercise samples exhibited enhanced relative abundance of *Pseudomonas*, *Gastranaerophilales*, *Barnesiella*, *Phascolarctobacterium*, and *Butyribrivio* (*p* < 0.01) (Figure 5B). Absolute log2FoldChange value represents the magnitude of the difference in relative abundance.

#### 3.1.7. Microbial Correlations with Emotional Well-Being and Nutrient Intake

Spearman’s correlational analysis was used to investigate whether emotional well-being or key nutrients (kcals, protein, fiber, total, omega-3, omega-6, and saturated fats) were correlated with alpha diversity. Nutrients were chosen based on their foundational role in dietary intake, known ability to shape the microbiota, or because they demonstrated significant differences in average intake between time points. Emotional well-being was not correlated with alpha diversity metrics at any time point. Similarly, no significant correlations were found for the nutrients.

To investigate the potential influence of nutrient intake on beta diversity, Adonis analysis was completed. The analysis was based on Weighted UniFrac distance matrix data across all three time points for all participants. When samples were categorized by allocating participants into three equal groups (low, medium, high intake) according to numerically ordered total fat values, the fat intake grouping demonstrated a significant 13.7% contribution to variance seen in beta diversity between samples (*p* = 0.034). The low total fat intake group averaged 55.6 ± 6.0 g/day, the medium group averaged 94.6 ± 2.2 g/day, and the high intake group averaged 138.3 ± 9.0 g/day. No other nutrients contributed significantly to variance in gut microbial communities.

#### 3.1.8. FMT Donor Choice for the Germ-Free Murine Study

**Figure 6:**
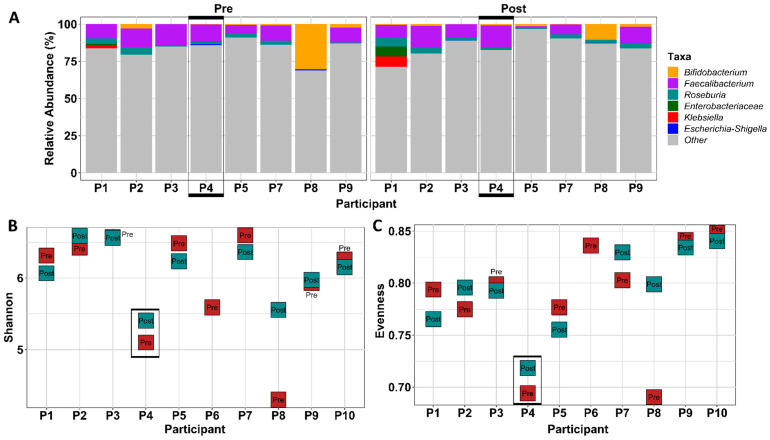
Fecal microbiota transplant (FMT) donor selection informed by relative abundance and alpha diversity. Relative abundance of three health-associated and three inflammation-associated genera were analyzed. Participant four exhibited an increase in *Faecalibacterium* from baseline to 12 weeks (ns) (**A**). Alpha diversity as measured by Shannon (**B**) and Evenness (**C**) indices increased from baseline to 12 weeks in participant four (ns).

Samples from a single participant at baseline and 12 weeks (post-exercise intervention) were selected as FMT donor material to use in the germ-free mouse study. Although there were no profound group shifts in microbiota due to exercise, likely in part due to our low sample size, we examined individual responses to exercise to select a donor participant who showed a positive microbial response to exercise. A beneficial response was defined by us as an increase in alpha diversity accompanied by an increase in health-associated gut microbiota and/or decrease in inflammation-associated gut microbiota. Participant 4 met the defined criteria and was selected as the FMT donor. Figure 6A shows that relative abundance of *Faecalibacterium* and *Roseburia* increased from baseline to week 12 in samples of participant 4. Although not statistically significant, Figure 6B,C show the increase in alpha diversity between baseline and week 12 in samples of participant 4 as measured by Shannon index and Pielou’s evenness index. Taken together, participant 4′s pre- and post-exercise samples indicate a promising gut microbial response to exercise and were chosen for investigation of the potential physiological effects of this response in a germ-free mouse model. Characteristics of the donor participant were 59 years of age, BMI = 25.1 kg/m^2^, calorie intake of 2393 kcal/d at baseline and 2484 kcal/d at week 12, similar FACT-G total score at baseline and 12 weeks, and an increase in MET hours per week from 9 to 13.5 with resistance exercise of 60 min/week at week 12 compared to 0 min/week at baseline. The donor attended 75% of the exercise classes, had received surgery, chemotherapy, and radiation, and reported regular drinking and previous smoker status.

### 3.2. Results of the Germ-Free Mouse Study Investigating the Impact of Exercise-Responsive Gut Microbiota in a Murine Model of Breast Cancer Treatment

#### 3.2.1. Fluid Intake

Fluid intake was recorded over the course of the study for the OFS and non-OFS groups since the oligofructose supplement was delivered dissolved in water. Fluid intake did not differ between mice receiving the oligofructose solution (6.53 ± 0.42 mL/mouse/day) and those who had water (5.84 ± 0.38 mL/mouse/day) (*p* = 0.192).

#### 3.2.2. Tumor Volumes Indicate Post-Exercise Microbiota-Related Benefit

**Figure 7:**
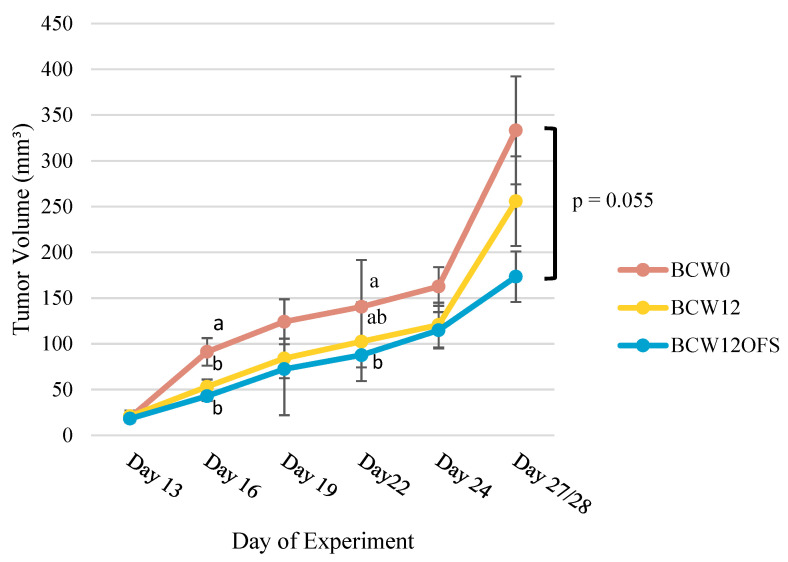
Tumor volume over time. Average tumor volume at each measurement time point is plotted for each group. BCW0 (*n* = 10), BCW12 (*n* = 12), BCW12OFS (*n* = 12). Average volume differed significantly between groups on day 16 and day 22. Values without a common superscript are significantly different (*p* < 0.05; i.e., ‘a’ is different from ‘b’ but ‘ab’ is not different from ‘a’ or ‘b’). Data are presented as mean ± SEM.

The results from analysis of caliper measurements to assess tumor volume over the course of the study are presented in Figure 7. Measurements were taken on days 13, 16, 19, 22, 24, and at endpoint which fell on either day 27 or 28. Overall, average tumor volume was consistently greater in the BCW0 mice compared to BCW12 and BCW12OFS mice. There was a significant difference in tumor volume between BCW0 and BCW12 (*p* = 0.034) as well as between BCW0 and BCW12OFS (*p* = 0.006) on day 16 of the study. On day 22, tumor volume was significantly smaller in the BW12OFS group compared to BCW0 (*p* = 0.043). At the endpoint, there was a trend (*p* = 0.055) for tumor volume to be lower in BCW12OFS compared to BCW0. Results indicate that post-exercise microbiota colonization resulted in smaller tumor volumes, and oligofructose supplementation enhanced this effect.

#### 3.2.3. Mouse Gut Microbial Composition Differs Significantly across Groups

**Figure 8:**
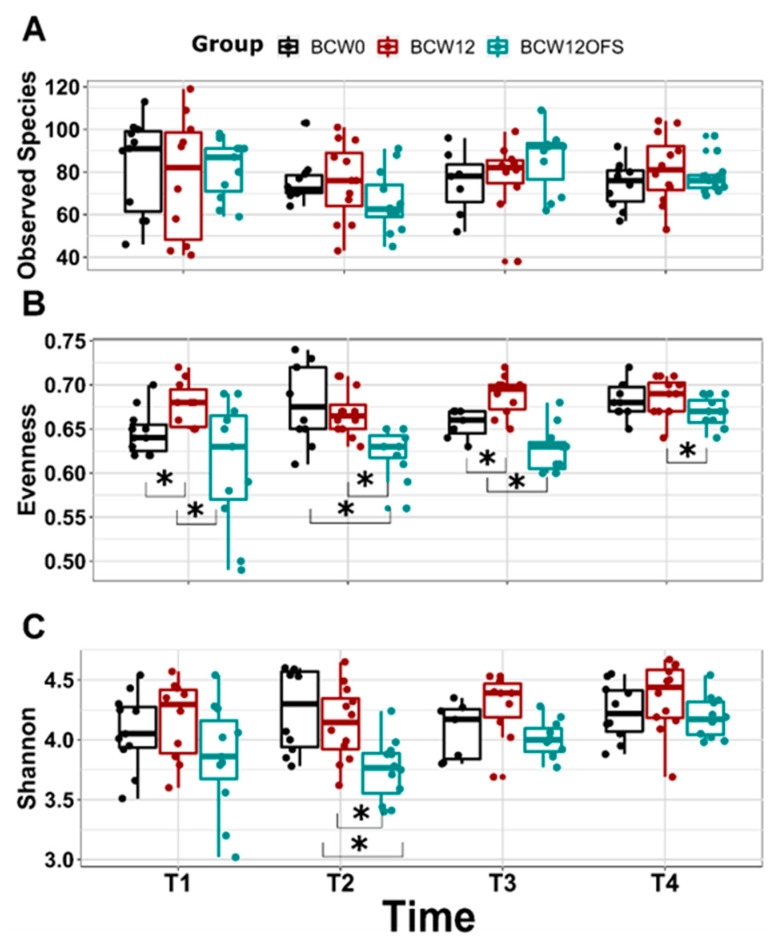
Mouse gut microbial alpha diversity across time points. Alpha diversity metrics measured at T1 (day 5—post-FMT), T2 (day 13—post tumor cell injection), T3 (day 22—post paclitaxel), and T4 (day 27/28—euthanasia) and compared between groups using Kruskal-Wallis pairwise tests (A-C). Observed Species did not differ significantly between groups (**A**). Evenness differed significantly between groups at each timepoint (**B**). Shannon diversity differed significantly between groups at T2 (day 13) (**C**). * Indicates a significant difference (*p* < 0.05). Data are presented as ± SEM and each dot represents an individual mouse’s results. BCW0 (*n* = 10), BCW12 (*n* = 12), BCW12OFS (*n* = 12).

Values of Alpha diversity, as measured by observes species, Shannon index, and Pielou’s evenness index, are presented in Figure 8. No significant difference in observed species was found between groups at any of the four time points (Figure 8A). On day 5, Pielou’s evenness was significantly lower in BCW12OFS compared to BCW12 (*p* = 0.02) (Figure 8B) and lower in BCW0 compared to BCW12 (*p* = 0.014) (Figure 8B). On day 13, which fell after tumor cell injection, BCW12OFS maintained significantly lower evenness than BCW12 (*p* < 0.001) and BCW0 (*p* = 0.006), while the difference between BCW0 and BCW12 was no longer significant (Figure 8B). Post-chemotherapy treatment on day 22, evenness was once again significantly lower in BCW12OFS compared to both BCW12 (*p* < 0.001) and BCW0 (*p* = 0.021) (Figure 8B), and BCW0 was lower than BCW12 (*p* = 0.011) (Figure 8B). At endpoint, evenness became more similar across groups. However, BCW12OFS mice remained lower than BCW12 mice (*p* = 0.015) (Figure 8B). Shannon diversity differed post tumor cell injection with the BCW12OFS group exhibiting lower Shannon diversity compared to both BCW12 (*p* = 0.005) and BCW0 (*p* = 0.002) (Figure 8C), which was no longer seen on day 22 and at endpoint.

**Figure 9:**
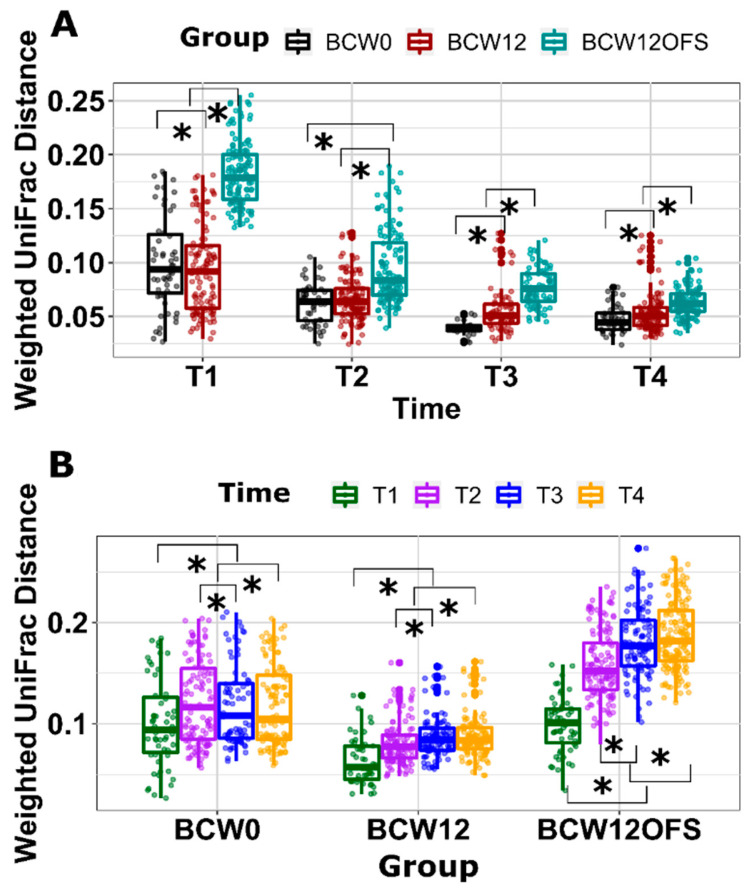
Mouse gut microbial beta diversity across time points. Beta diversity as measured by Weighted UniFrac Distance at T1 (day 5—post-FMT), T2 (day 13—post tumor cell injection), T3 (day 22—post paclitaxel), and T4 (day 27/28—euthanasia) and analyzed using ANOSIM to detect significant between-group community differences at each time are presented (**A**). Weighted UniFrac Distance analyzed with ANOSIM to detect community differences over time within each group are also presented (**B**). * indicates a significant difference (*p* < 0.05). Data are presented as ± SEM.

Mouse beta diversity is presented in Figure 9. Figure 9A depicts beta diversity between groups at each time point while Figure 9B shows the within-group comparisons across all time points. Figure 9A shows that the gut microbial community in BCW12OFS mice differed significantly compared to BCW0 and BCW12 at each of the four time points (*p* < 0.05). The gut microbial community in BCW12 mice differed from BCW0 at all time points (*p* < 0.05) except day 13 (T2) (Figure 9A). A significant difference in beta diversity across all four times is evident within each group as displayed in Figure 9B (*p* < 0.05), indicating that the microbial community was altered from baseline to when tumor cells were injected to when chemotherapy was administered to the end of the study.

**Figure 10:**
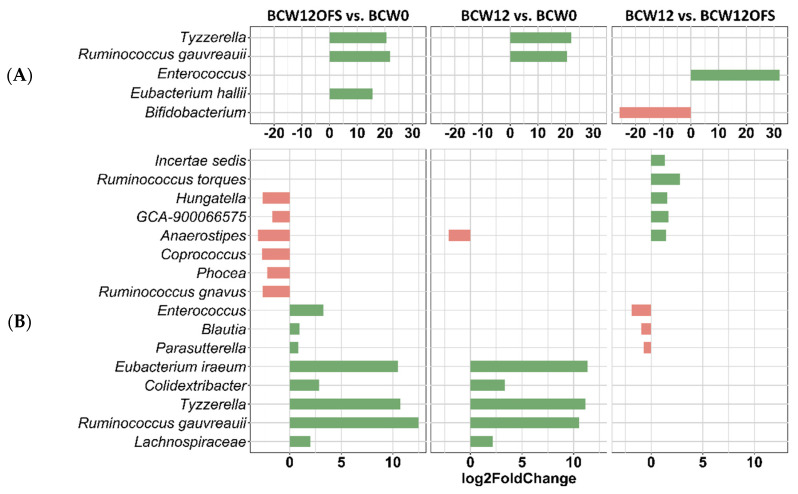
Mouse gut microbiota differential abundance analyses. Taxa found to be differentially abundant between groups are represented by log2FoldChange from DESeq2 analysis. The group serving as the base for comparison comes after “vs.” (i.e., BCW12OFS vs. BCW0 is showing values for the bacteria in BCW12OFS compared to BCW0). Positive log2FoldChange indicates greater relative abundance, while a negative value indicates lesser relative abundance. Significantly differential abundance for each comparison at day 13 are presented (**A**). Significantly differential abundance for each comparison at day 22 are also presented (**B**). Only differences with *p* < 0.001 from DESeq2 analysis are represented. BCW0 (*n* = 10), BCW12 (*n* = 12), BCW12OFS (*n* = 12).

Differential abundance analysis with DESeq2 was performed to determine differences in taxonomic composition at day 13, which was after tumor cell injection (Figure 10A), and at day 22, which was after chemotherapy conclusion (Figure 10B). Differentially abundant genera with *p* < 0.001 are displayed. On day 13, BCW12OFS had significantly greater abundance of *Tyzerella*, *Ruminococcus gauvreauii*, and *Eubacterium hallii* compared to BCW0 (Figure 10A). At the same time point, BCW12 also showed enrichment in *Tyzzerella* and *Ruminococcus gauvreauii* compared to BCW0 (Figure 10A). BCW12 had significantly greater abundance of *Enterococcus* and decreased abundance of *Bifidobacterium* compared to BCW12OFS at day 13 (Figure 10A). On day 22, following chemotherapy, the greatest number of bacteria was found to be differentially abundant between groups (Figure 10B). BCW12OFS mice exhibited greater *Enterococcus*, *Blautia*, *Parasutterella*, *Eubacterium iraeum*, *Colidextribacter*, *Tyzzerella*, *Ruminococcus gauvreauii*, and *Lachnospiraceae* alongside lesser abundance of *Hungatella*, *GCA-900066575*, *Anaerostipes*, *Coprococcus*, *Phocea*, and *Ruminococcus gnauvus* compared to BCW0 (Figure 10B). On day 22, BCW12 similarly presented with higher abundance of *Eubacterium iraeum*, *Colidextribacter*, *Tyzzerella*, *Ruminococcus gauvreauii*, and *Lachnospiraceae* compared to BCW0 (Figure 10B). Compared to BCW12OFS, the BCW12 group exhibited greater abundance of *Incertae_Sedis*, *Ruminococcus torques*, *Hungatella*, *GCA-900066575*, and *Anaerostipes*, coinciding with significantly lower abundance of *Enterococcus*, *Blautia*, and *Parasutterella* (Figure 10B).

#### 3.2.4. Ileal and Colonic Tight Junction Proteins

RT-PCR analysis was conducted to assess mRNA levels of tight junction proteins involved in maintaining intestinal barrier function. There were no differences in ileal or colonic expression of zonula occludens (ZO-1), occludin, and claudin-3 (data not shown).

#### 3.2.5. Tumor and Serum Cytokine Levels

**Figure 11:**
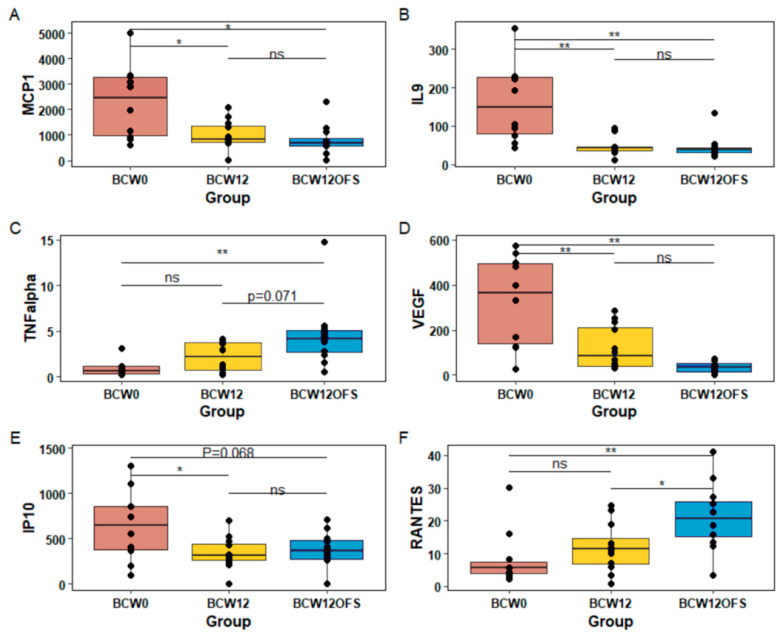
Levels of key tumor cytokines. Tumor cytokine levels as measured by multiplex assay which differed significantly between groups are presented (**A**–**F**). These include levels of MCP-1 (**A**), IL-9 (**B**), TNFα (**C**), VEGF (**D**), IP-10 (**E**), and RANTES (**F**). * Indicates a significant difference of *p* < 0.05 and ** indicates a significant difference of *p* < 0.01. Data are presented as ± SEM and each point represents an individual mouse’s results. BCW0 (*n* = 10), BCW12 (*n* = 12), BCW12OFS (*n* = 12) for tumor cytokines and BCW0 (*n* = 9), BCW12 (*n* = 11), BCW12OFS (*n* = 12) for serum. ns: not significant.

Given that inflammation is a critical component of tumor progression [72] and the microbiota can affect inflammation in the host [73], we examined tumor and serum cytokine levels. Monocyte chemoattractant protein-1 (MCP-1) levels were higher in BCW0 tumors compared to BCW12 (*p* = 0.007) and BCW12OFS (*p* = 0.002), which did not differ from each other (Figure 11A). Similarly, tumor interleukin-9 (IL-9) levels were higher in BCW0 mice compared to both BCW12 and BCW12OFS mice (*p* < 0.001) (Figure 11B). Tumor necrosis factor alpha (TNFα) levels were higher in BCW12OFS mice compared to BCW0 mice (*p* = 0.009) and approached significance compared to BCW12 (*p* = 0.071) (Figure 11C). Tumor levels of vascular endothelial growth factor (VEGF) were significantly higher in BCW0 mice compared to both BCW12 (*p* = 0.001) and BCW12OFS (*p* < 0.001) (Figure 11D). Interferon gamma-induced protein-10 (IP-10) levels were higher in BCW0 compared to BCW12 tumors (*p* = 0.036) and approached significance in comparison to BCW12OFS tumor levels (*p* = 0.068, Figure 11E). Levels of tumor RANTES (Regulated upon Activation, Normal T Cell Expressed and Presumably Secreted) were higher in BCW12OFS mice compared to both BCW0 mice (*p* = 0.005) and BCW12 mice (*p* = 0.037; Figure 11F). No differences were detected for tumor levels of Eotaxin, G-CSF (granulocyte colony stimulating factor), GM-CSF (granulocyte macrophage colony stimulating factor), interferon gamma (IFNy), KC (keratinocytes-derived chemokine), MIP-1α (macrophage inflammatory protein), MIP-2, IL-1α, IL-1β, IL-6, IL-5, IL-4, IL-10, IL-12p40, IL-12p70, IL-13, IL-15, LIF (leukemia inhibitory factor), M-CSF, or MIG (monokine induced by gamma interferon) (data not shown).

Serum cytokines were far more refractory to change compared to tumor cytokine levels. There were no significant differences in serum levels of any of the cytokines apart from serum levels of LIX (lipopolysaccharide-inducible CXC chemokine (CXCL5)) which were significantly higher in BCW12 mice compared to BCW0 (*p* < 0.05).

Five cytokines (IL-10, KC, LIF, MIP-2, and VEGF) involved in tumorigenesis, angiogenesis, and chemoattraction of immune cells were found to have significant positive correlations with tumor volume (Table 8).

## 4. Discussion

The gut microbiota exerts a robust influence on metabolism, energy harvest, the immune system, and inflammation [5,36,74,75]. The present study was designed to investigate the effects of an exercise intervention on the gut microbiota of women who had undergone chemotherapy for breast cancer and to use FMT in germ free mice to explore mechanisms by which an exercise-responsive gut microbiota might influence tumor growth and response to chemotherapy treatment. Overall, we demonstrate that the 12-week exercise intervention did not significantly alter the gut microbiota community structure in our small clinical cohort. However, in the FMT experiment, the post-exercise gut microbiota from an individual who demonstrated a positive microbial response to exercise did significantly alter the tumor microenvironment and gut microbial response to chemotherapy, especially when combined with oligofructose supplementation.

In healthy adults, greater cardiorespiratory fitness and higher reported physical activity levels have been associated with greater microbial alpha diversity [76]. Although a previous study demonstrated promising positive correlations between cardiorespiratory fitness and alpha diversity metrics in breast cancer survivors [45], alpha diversity as measured by Pielou’s evenness index, Shannon index, and observed species did not differ significantly between pre- and post- exercise time points in our participants. However, some participants demonstrated a trend toward increased alpha diversity between 0 and 12 weeks. This variability is not surprising given the “individualized and varying response” to exercise previously reported between lean individuals and those with obesity [77,78]. In our study, BMIs ranged from underweight to obese which likely influenced the alpha diversity results [79]. Although our sample size is too small to draw concrete conclusions, it is likely that some individuals could have a more exercise-responsive gut microbiota than others based on environmental, genetic, or epigenetic factors known to shape the gut microbiota [80]. It is also possible that our exercise intervention was not of sufficient intensity and/or duration to generate the magnitude of increase in cardiorespiratory fitness necessary to produce the shifts that have been seen in other studies [40]. Despite the lack of change in overall community structure metrics, such as alpha and beta diversity, shifts in select bacterial taxa have been observed with exercise [45,81,82,83].

In our study, we examined the relative abundance of three health-associated and three inflammation-associated bacteria. *Bifidobacterium*, *Faecalibacterium*, and *Roseburia* are SCFA-producing bacteria known for competitive exclusion of pathogens, participation in nutrient metabolism, and support of colonocyte function [65,66,68]. In contrast, *Enterobacteriaceae*, *Klebsiella*, and *Escherichia-Shigella* are known as opportunistic pathogens that have been associated with inflammatory conditions [69,84,85]. Carter et al. observed that breast cancer survivors with higher cardiorespiratory fitness had a greater relative abundance of *Faecalibacterium* [45], which aligns with gut microbial findings in participants 2, 4, and 9 who appeared to be more exercise-responsive in the present study. This trend is also in alignment with the increased relative abundance of *Faecalibacterium prausnitzii* and *Roseburia hominis* observed in women exercising 150-min or more per week compared to sedentary women [83]. *Faecalibacterium rodentium* attenuated the accelerated breast tumor growth caused by antibiotic treatment in a mouse model, highlighting the potential benefit of this genera [86].

Eight genera outside of our chosen markers were found to be differentially abundant in the survivors between baseline and 12 weeks. This suggests that despite the lack of statistically significant shifts in overall community structure, exercise influenced specific bacteria within the gut microbiota of participants. Pre-exercise samples exhibited higher relative abundance of *Pseudomonas*, *Gastranaerophilales*, *Barnesiella*, *Phascolarctobacterium*, and *Butyrivibrio* compared to post-exercise samples. Members of *Pseudomonas* are opportunistic pathogens that are not abundant in healthy individuals and are known to cause infection in individuals with cancer [87], so a decrease may be beneficial. The relative abundance of *Dialister*, *Oscillospiraceae*, and *Paraprevotella* increased between baseline and 12 weeks with the ACE program. The observed increased abundance of *Paraprevotella* with exercise is in alignment with findings from Bressa et al. which indicated that active women had greater abundance of *Paraprevotella* compared to sedentary women [83]. These bacteria may be particularly exercise responsive. Although significant taxonomic changes associated with the exercise intervention are limited, they may influence the metabolic potential of the community and the intestinal environment in ways that could benefit host health.

Outcomes from our mouse FMT study indicate that groups colonized with post-exercise gut microbiota (BCW12 and BCW12OFS) exhibited a pattern of smaller tumor volume compared to BCW0. Previous studies in mice have linked exercise that is performed pre-tumor cell injection and post-tumor injection with suppressed breast tumor growth [88,89]. These studies posit that the tumor-suppressive effects of exercise were mediated by alterations in relevant circulating immune cells that are widely known to occur in response to acute exercise [90]. Here, it is demonstrated that exercise may also beneficially alter gut microbiota in a way that promotes tumor suppression independent from the direct, acute effect of the exercise (since our donor performed the exercise and not the mice). Furthermore, the group that received prebiotic oligofructose exhibited the smallest tumor volume and the greatest number of statistically significant time points compared to the pre-exercise group. The anti-tumor effect of oligofructose was previously shown in rats and mice, whereby tumor incidence was decreased and oligofructose acted synergistically in combination with various chemotherapeutics [91,92].

High alpha diversity is often associated with gut microbial health. However, here, the BCW12OFS mice exhibited a trend towards or significantly decreased alpha diversity compared to the other groups at most time points. This is likely due to oligofructose’s ability to support the proliferation of select beneficial bacteria [53,93] which may in turn dominate the community structure and competitively exclude other bacteria, thus reducing alpha diversity. A decrease in alpha diversity with prebiotic consumption has been noted in a human intervention trial as well and was associated with improved obesity-related health outcomes [94].

Beta diversity results indicate that communities differed significantly between all groups at each time point. In the recipient mice, it is evident that communities differed between the baseline gut microbiota (FMT for BCW0 mice) and the post-exercise gut microbiota (FMT for BCW12 and BCW12OFS mice) from participant 4. Although gut microbial beta diversity continued to differ significantly throughout the study, groups tended to cluster more closely over time. It is plausible that the injection of the breast tumor cells altered the gut microbiota, which would support findings in humans demonstrating that disease-specific community differences strongly associated with either individuals with breast cancer or healthy individuals [28]. Additionally, significant gut microbial community differences between mice receiving no treatment and those treated with Paclitaxel in murine models of breast cancer have previously been reported [95], so the communities may become more similar at day 22 and at the endpoint due to the effects of the cytotoxic exposure alongside tumor progression. In women with breast cancer, researchers found that beta diversity differed significantly between groups based on tumor size (T1 vs. T2&T3), indicating that progression could influence the gut microbial community over time [96]. These findings provide further evidence that breast tumor initiation, progression, and chemotherapeutic treatment with Paclitaxel are influential environment-modifying events associated with alterations in the gut microbial community.

Results were presented for differential taxonomic abundances at key time points which included day 13, following tumor cell injection, and day 22, following completion of Paclitaxel treatment. These time points were chosen for in-depth analysis due to previous studies indicating that tumor presence and Paclitaxel treatment both alter the gut microbiota [28,95,97], and the greatest number of bacteria appeared to be differentially abundant between groups at these time points. On day 13, *Tyzzerella*, *Ruminococcus gauvreauii*, and *Eubacterium hallii* were significantly more abundant in BCW12OFS mice compared to BCW0. Increased relative abundance of *Eubacterium hallii* in BCW12OFS could be considered positive considering its ability to metabolize glucose, fermentative products, and metabolic products into butyrate or propionate which could support intestinal barrier health and host immunity [98]. *Eubacterium hallii* are also capable of supporting host health by producing essential vitamin B12 [98].

Compared to BCW0, gut microbiota of BCW12 mice were also enriched in *Tyzzerella* and *Ruminococcus gauvreauii* after tumor cell injection, but not *Eubacterium halli*. This suggests that oligofructose supplementation was responsible for the uniquely increased *Eubacterium halli* in BCW12OFS mice while the other bacteria resulted from the exercise-responsive gut microbiota colonization. On day 13, BCW12OFS was enriched in *Bifidobacterium* with a lesser abundance of *Enterococcus* compared to BCW12. Oligofructose is known to support proliferation of beneficial *Bifidobacterium* [93], and inulin, a longer chain prebiotic, has been reported to diminish enterococci in conjunction with the bifidogenic effect [99]. *Bifidobacterium* are beneficial in their immunomodulatory and anti-inflammatory activity [65,100]. BCW12 and BCW12OFS gut microbiota were enriched for health-supporting bacteria compared to BCW0 after tumor cell injection, with additional benefits seen with oligofructose supplementation.

On day 22, following chemotherapy treatment, more genera were differentially abundant between groups compared to day 13, suggesting that the cytotoxic chemotherapy Paclitaxel influenced the gut microbiota more significantly than breast tumor presence alone. Following Paclitaxel administration, 14 bacterial groups differed in BCW12OFS mice compared to BCW0 mice. For example, OFS enriched *Parasutterella* which has previously been reported to increase in abundance with inulin supplementation, resulting in greater presence of tumor infiltrating lymphocytes at the tumor site in a murine model of melanoma [16]. *Lachnospiraceae* was also increased, which may play an important role in anti-breast tumor immunity specifically, as its abundance was reported to be decreased in non-responders to Trastuzumab treatment for HER-2 positive breast cancer [101] and in pre-menopausal individuals with breast cancer in general [29].

Tumor and serum cytokines were analyzed to investigate a potential mechanism for the tumor volume differences between groups and to provide insight on the tumor microenvironment. Circulating cytokine levels also serve as useful biomarkers for tumor prognosis in human breast cancer [102]. Interestingly, most serum cytokines were not altered significantly between groups. BCW0 mice exhibited significantly higher intra-tumoral levels of MCP-1(CCL2), IL-9, and VEGF compared to BCW12 and BCW12OFS mice whose levels did not differ significantly from each other. This indicates that the pre-exercise gut microbiota was a potential driver of the increased levels in BCW0.

MCP-1(CCL2) is a chemoattractant cytokine that recruits monocytes from the blood to the tumor site where they then differentiate into macrophages [103]. Depending on environmental factors, monocytes will become more immune-activating (M1) or immune-suppressing (M2) tumor associated macrophages [103,104]. MCP-1(CCL2) could therefore play a role in immune-suppression, allowing for greater tumor proliferation in the BCW0 mice compared to the other groups. Lam et al. (2021) demonstrated that favorable gut microbiota mediate polarization of monocytes in the tumor microenvironment via microbiota-derived molecules such as the cyclic dinucleotide c-di-AMP. The molecules can activate stimulator of interferon genes (STING) to produce IFN Is (IFNα and IFNβ), beneficially modulating the tumor immune environment by regulating monocyte to macrophage polarization and influencing natural killer and dendritic cell activity which contributes to anti-tumor immunity [9]. Increased secretion of RANTES (CCL5) by natural killer cells in the tumor microenvironment is noted in this pathway [9], and RANTES was found to be elevated in BCW12OFS tumors. The pathway is enhanced by the microbiota of mice fed a high fiber diet [9], which presents a possible microbiota-mediated mechanism to explain the decreased tumor volume with oligofructose supplementation reported here. This is a promising potential mechanism. However, other reported mechanisms for microbial enhancement of chemotherapy response, such as immune-enhancing bacterial translocation to lymphoid organs, cannot be ruled out as this was not investigated in the present study [8,34].

VEGF is secreted by breast cancer cells in response to hypoxia to stimulate the angiogenesis necessary for continued cell proliferation and tumor growth [105] and contributes to breast cancer’s metastatic potential and apoptosis resistance [106,107]. The elevated levels in the BCW0 tumor microenvironment reflect increased growth and metastatic potential which are negative for overall prognosis. The significantly decreased intra-tumoral VEGF levels observed in post-exercise microbiota mice may be indicative of improved Paclitaxel treatment response or lower vascularity potential due to a possible gut microbial influence on the tumor microenvironment.

IP-10 (CXCL10) was higher in BCW0 tumor tissue compared to BCW12 and demonstrated that trend compared to BCW12OFS. Tumoral IP-10 (CXCL10) has been correlated with tumor stage and lymphoid metastasis in women with breast cancer, with higher levels indicating poorer prognosis [108]. IP-10 (CXCL10) has also been demonstrated to induce cell proliferation, migration, and epithelial to mesenchymal transition in MCF-7 and MDA-MB-231 breast cancer cell lines [109], which would contribute to a more aggressive profile supportive of greater tumor volume in BCW0 mice.

Several of the cytokine changes observed in the present study contribute evidence toward a more angiogenic, immuno-suppressed tumor microenvironment being observed in the BCW0 mice and a more active anti-tumor immune environment in the BCW12, and especially BCW12OFS, mice. Evidence of immune activity stimulation within the tumor microenvironment provides a potential mechanism for the gut microbiota-potentiated benefit of exercise and prebiotic supplementation during breast cancer treatment in this model.

## 5. Limitations

There are several limitations of this study which must be considered. The clinical portion of the study would have benefitted from a larger sample size, a control group, and additional metrics, such as a body-composition analysis. Although BMI is used throughout medical and nutrition research, it does not take body composition into account and is not always an accurate measure of health. The murine study only included tumor cytokine analysis to interrogate the conditions of the tumor microenvironment between groups. Additional histological and flow cytometry analysis on the tumor tissue would have provided insight into other microenvironment indices, such as tumor cell viability/proliferation and immune cell infiltration.

## 6. Conclusions

The human portion of the study indicated a limited effect of the exercise intervention on participant gut microbiota which was primarily distinguished by the differential relative abundance of several genera between baseline and the conclusion of the ACE program. Future clinical work would benefit from a larger cohort and a randomized controlled trial design. Despite the small sample size, select participants exhibited an increase in alpha diversity with exercise, which we hypothesize is indicative that some individuals will experience a greater gut microbial response to exercise compared to others. Exercise may serve as a possible intervention to attenuate treatment and breast cancer-associated gut microbial dysbiosis, but additional research is needed to clarify effective exercise modalities, frequencies, and durations. Although the shifts in gut microbiota in response to exercise in the survivors seemed minimal, FMT of a participant’s baseline and exercise responsive microbiota in a germ-free model of breast cancer resulted in significant differences in tumor volume, gut microbiota, and immunologically active tumor cytokines over time. Some of the effects, such as decreased tumor volume, decreased angiogenesis markers, and increased markers of Paclitaxel response in the tumor microenvironment, were enhanced by prebiotic oligofructose supplementation. Exercise and prebiotic supplementation appear to beneficially modulate anti-tumor immunity in part through favorable modification of the gut microbiota. Further research will be necessary to characterize the interaction between gut microbiota and the tumor microenvironment more completely. However, taken together, these results point to the benefit of exercise and prebiotic supplementation as adjuvant interventions.

## Figures and Tables

**Table 1:** Inclusion and Exclusion Criteria for Recruitment.

Inclusion Criteria	Exclusion Criteria
Biologically female	Intestinal diseases such as ulcerative colitis/Crohn’s
Clinically diagnosed with breast cancer	Body mass index (BMI) >35 kg/m^2^
Have undergone chemotherapy as a part of their treatment	Probiotic or prebiotic supplementation (this does not include consumption of foods which contain probiotic or prebiotic)
	History of major gastrointestinal surgery
	Regular consumption of >1 alcoholic beverage/day
	Pregnant or lactating
	Currently undergoing chemotherapy or immunotherapy

**Table 2:** Primer Sequences.

Gene	Forward Sequence	Reverse Sequence
ZO-1 (zonula occludens)	AGGGGCAGTGGTGGTTTTCTGGTTCTTTC	GCAGAGGTCAAAGTTCAAGGCTAAGAGG
Occludin	TCAGGGAATATCCACCTATCACTTCAG	CATCAGCAGCAGCCATGTACTCTTCAC
Claudin-3	CACCGCACCATCACCACTAC	CTTCCAGCCTAGCAAGCAGAC

**Table 3:** Demographic information on participants recruited from the Alberta Cancer Exercise (ACE) program (*n* = 10).

Demographic		Mean	Frequency	Percent
Age	Average Age (years)	57.9 ± 2.79		
	Under 65		8	80
	Over 65		2	20
Education	Some University		3	20
	Completed University		6	60
	Some Graduate School		1	10
Annual Income	Between $20,000–39,999		2	20
	Between $40,000–59,999		1	10
	Between $60,000–79,999		1	10
	Between $80,000–99,999		2	20
	Over $99,999		4	40
Ethnic Background	Britain		4	40
(May Report >1)	Western Europe		2	20
	Eastern Europe		4	40
	Northern Europe		3	30
	Southern Europe		2	20
	Asia		2	20
Smoking Status	Never		6	60
	Previously		4	40
Alcohol Consumption	Never		2	20
	Previously		1	10
	Occasionally		5	50
	Socially		1	10
	Regularly		1	10

**Table 4:** Clinical characteristics of the participants recruited from ACE.

Clinical Characteristic		Mean	Frequency	Percent
Completed Treatments	Chemotherapy		10	100
	Surgery		10	100
	Radiation Therapy		8	80
	Hormone Therapy		1	10
Current Treatments	Hormone Therapy		5	50
	Zoledronic Acid Infusions		1	10
Body mass index	Underweight BMI (<18.5)		1	10
	Healthy BMI (18.5–24.9)		2	20
	Overweight BMI (25–29.9)		4	40
	Obese BMI (≥30)		3	30

Body Mass Index, BMI.

**Table 5:** Godin’s Leisure Time Exercise Questionnaire.

Exercise Category & Week	MET Hours/Week	*p* Value 0 to 12 Weeks	*p* Value 12 to 24 Weeks	*p* Value 0 to 24 Weeks
Total 0	18.4 ± 4.2	0.002		
Total 12	33.6 ± 5.2		0.535	
Total 24	38.1 ± 7.7			0.030
Strenuous 0	4.7 ± 2.6	0.016		
Strenuous 12	14.2 ± 3.8		0.280	
Strenuous 24	20.5 ± 7.0			0.075
Moderate 0	8.8 ± 2.2	0.301		
Moderate 12	12.2 ± 3.2		0.452	
Moderate 24	10.2 ± 1.9			0.626
Mild 0	4.8 ± 0.8	0.072		
Mild 12	7.1 ± 1.5		0.891	
Mild 24	7.3 ± 1.5			0.150
	Minutes/week			
Resistance 0	34.4 ± 20.4	0.113		
Resistance 12	69.4 ± 22.0		0.767	
Resistance 24	63.3 ± 17.1			0.224
Flexibility 0	45.5 ± 11.5	0.276		
Flexibility 12	71.1 ± 21.3		0.816	
Flexibility 24	74.4 ± 16.1			0.078

Metabolic equivalent of task, MET.

**Table 6:** Dietary intake at baseline, 12 and 24 weeks.

Nutritional Measure	Time Point	Daily Average	Consecutive Time Point *p* Value
Calories (kcal)	Baseline	2069.3 ± 188.8	
	12 weeks	2260.2 ± 114.9	0.404
	24 weeks	1785.2 ± 196.9	0.017
Protein (g)	Baseline	79.5 ± 7.9	
	12 weeks	87.3 ± 5.0	0.352
	24 weeks	73.7 ± 6.9	0.125
Carbohydrate (g)	Baseline	232.3 ± 20.9	
	12 weeks	234.3 ± 10.8	0.936
	24 weeks	210.4 ± 21.2	0.359
Total Fat (g)	Baseline	92.6 ± 11.2	
	12 weeks	107.4 ± 9.0	0.338
	24 weeks	74.1 ± 12.9	0.012
Polyunsaturated Fat (g)	Baseline	17.7 ± 1.9	
	12 weeks	24.1 ± 3.2	0.244
	24 weeks	13.1 ± 2.0	0.004
Monounsaturated Fat (g)	Baseline	30.5 ± 3.5	
	12 weeks	38.1 ± 3.2	0.153
	24 weeks	24.6 ± 3.9	<0.001
Saturated Fat	Baseline	29.9 ± 3.9	
	12 weeks	36.0 ± 5.3	0.387
	24 weeks	22.6 ± 3.9	0.057
Fiber (g)	Baseline	23.9 ± 4.4	
	12 weeks	29.7 ± 4.9	0.352
	24 weeks	27.8 ± 6.2	0.478
Vitamin E (mg)	Baseline	8.7 ± 1.5	
	12 weeks	10.9 ± 1.3	0.365
	24 weeks	6.6 ± 1.2	0.029
Selenium (mcg)	Baseline	109.9 ± 8.9	
	12 weeks	121.5 ± 8.9	0.466
	24 weeks	100.9 ± 8.0	0.021

**Table 7:** FACT-G questionnaire results.

FACT-G Total Score (108 Max)	Timepoint	Consecutive Timepoint *p* Value	Median Score	Sample Size	Completion%
84.6 ± 5.2	Baseline		87.5	N = 10	100
84.9 ± 4.8	12 Weeks	0.869	92	N = 9	90
81.4 ± 5.3	24 Weeks	0.302	82	N = 9	90
**Well-being Category**	Timepoint	Categorical Score	Consecutive timepoint *p* value		Median Score
Physical	Baseline	23.9 ± 1.3			25.0
	12 weeks	24.7 ± 0.9	0.560		24.0
	24 weeks	24.9 ± 0.7	0.816		25.0
Social	Baseline	23.7 ± 1.2			23.5
	12 weeks	23.2 ± 1.4	0.688		22.0
	24 weeks	22.4 ± 1.4	0.065		21.0
Emotional	Baseline	16.4 ± 0.9			17.0
	12 weeks	16.2 ± 1.1	0.327		21.0
	24 weeks	14.9 ± 1.6	0.291		14.0
Functional	Baseline	21.2 ± 2.0			20.0
	12 weeks	18.3 ± 1.5	0.091		18.0
	24 weeks	20.4 ± 1.7	0.082		21.0

**Table 8:** Significant correlations between tumor volume and cytokine levels.

Cytokine	Spearman’s Correlation Coefficient	Significance
IL-10	0.434	0.030
KC	0.530	0.001
LIF	0.500	0.004
MIP-2	0.529	0.002
VEGF	0.392	0.022

## Data Availability

The data presented in this study are available on request from the corresponding author. The data are not publicly available because this sub-study is part of a larger study.

## References

[1] Noce A., Marrone G., Daniele F.D., Ottaviani E., Jones G.W., Bernini R., Romani A., Rovella V. (2019). Impact of Gut Microbiota Composition on Onset and Progression of Chronic Non-Communicable Diseases. Nutrients.

[2] Vivarelli S., Salemi R., Candido S., Falzone L., Santagati M., Stefani S., Torino F., Banna G.L., Tonini G., Libra M. (2019). Gut microbiota and cancer: From pathogenesis to therapy. Cancers.

[3] Scott A.J., Alexander J.L., Merrifield C.A., Cunningham D., Jobin C., Brown R., Alverdy J., Keefe S.J.O., Gaskins H.R., Teare J. (2019). International Cancer Microbiome Consortium consensus statement on the role of the human microbiome in carcinogenesis. Gut.

[4] Mager L.F., Burkhard R., Pett N., Cooke N.C.A., Brown K., Ramay H., Paik S., Stagg J., Groves R.A., Gallo M. (2020). Microbiome-derived inosine modulates response to checkpoint inhibitor immunotherapy. Science.

[5] Frosali S., Pagliari D., Gambassi G., Landolfi R., Pandolfi F., Cianci R. (2015). How the Intricate Interaction among Toll-Like Receptors, Microbiota, and Intestinal Immunity Can Influence Gastrointestinal Pathology. J. Immunol. Res..

[6] Soares P.M.G., Mota J.M.S.C., Souza E.P., Justino P.F.C., Franco A.X., Cunha F.Q., Ribeiro R.A., Souza M.H.L.P. (2013). Inflammatory intestinal damage induced by 5-fluorouracil requires IL-4. Cytokine.

[7] Karpinets T.V., Prieto P.A., Vicente D., Hoffman K., Wei S.C., Cogdill A.P., Zhao L., Hudgens C.W., Hutchinson D.S., Manzo T. (2018). Gut microbiome modulates response to anti—PD-1 immunotherapy in melanoma patients. Science.

[8] Viaud S., Saccheri F., Mignot G., Yamazaki T., Daillère R., Hannani D., Enot D.P., Pfirschke C., Engblom C., Pittet M.J. (2013). The Intestinal Microbiota Modulates the Anticancer Immune Effects of Cyclophosphamide. Science.

[9] Lam K.C., Araya R.E., Huang A., Chen Q., Di Modica M., Rodrigues R.R., Lopès A., Johnson S.B., Schwarz B., Bohrnsen E. (2021). Microbiota triggers STING-type I IFN-dependent monocyte reprogramming of the tumor microenvironment. Cell.

[10] Kumar J., Rani K., Datt C. (2020). Molecular link between dietary fibre, gut microbiota and health. Mol. Biol. Rep..

[11] Gibson G.R., Hutkins R., Sanders M.E., Prescott S.L., Reimer R.A., Salminen S.J., Scott K., Stanton C., Swanson K.S., Cani P.D. (2017). Expert consensus document: The International Scientific Association for Probiotics and Prebiotics (ISAPP) consensus statement on the definition and scope of prebiotics. Nat. Rev. Gastroenterol. Hepatol..

[12] Zmora N., Suez J., Elinav E. (2019). You are what you eat: Diet, health and the gut microbiota. Nat. Rev. Gastroenterol. Hepatol..

[13] Mailing L.J., Allen J.M., Buford T.W., Fields C.J., Woods J.A. (2019). Exercise and the Gut Microbiome: A Review of the Evidence, Potential Mechanisms, and Implications for Human Health. Exerc. Sport Sci. Rev..

[14] Mohr A.E., Jäger R., Carpenter K.C., Kerksick C.M., Purpura M., Townsend J.R., West N.P., Black K., Gleeson M., Pyne D.B. (2020). The athletic gut microbiota. J. Int. Soc. Sports Nutr..

[15] Sheflin A.M., Whitney A.K., Weir T.L. (2014). Cancer-Promoting Effects of Microbial Dysbiosis. Curr. Oncol. Rep..

[16] Li Y., Elmén L., Segota I., Xian Y., Tinoco R., Feng Y., Fujita Y., Segura Muñoz R.R., Schmaltz R., Bradley L.M. (2020). Prebiotic-Induced Anti-tumor Immunity Attenuates Tumor Growth. Cell Rep..

[17] Betof A.S., Lascola C.D., Weitzel D., Landon C., Scarbrough P.M., Devi G.R., Palmer G., Jones L.W., Dewhirst M.W. (2015). Modulation of Murine Breast Tumor Vascularity, Hypoxia, and Chemotherapeutic Response by Exercise. J. Natl. Cancer Inst..

[18] Spiliopoulou P., Gavriatopoulou M., Kastritis E., Dimopoulos M., Terzis G. (2021). Exercise-Induced Changes in Tumor Growth via Tumor Immunity. Sports.

[19] Clinton S.K., Giovannucci E.L., Hursting S.D. (2020). The World Cancer Research Fund/American Institute for Cancer Research Third Expert Report on Diet, Nutrition, Physical Activity, and Cancer: Impact and Future Directions. J. Nutr..

[20] Ecker B.L., Lee J.Y., Sterner C.J., Solomon A.C., Pant D.K., Shen F., Peraza J., Vaught L., Mahendra S., Belka G.K. (2019). Impact of obesity on breast cancer recurrence and minimal residual disease. Breast Cancer Res..

[21] Lee K., Kruper L., Dieli-conwright C.M., Mortimer J.E. (2019). The Impact of Obesity on Breast Cancer Diagnosis and Treatment. Curr. Oncol. Rep..

[22] Pierce J.P., Stefanick M.L., Flatt S.W., Natarajan L., Sternfeld B., Al-delaimy W.K., Thomson C.A., Kealey S., Hajek R., Parker A. (2008). Greater Survival After Breast Cancer in Physically Active Women with High Vegetable-Fruit Intake Regardless of Obesity. J. Clin. Oncol..

[23] Cannioto R.A., Hutson A., Dighe S., McCann W., McCann S.E., Zirpoli G.R., Barlow W., Kelly K.M., DeNysschen C.A., Hershman D.L. (2021). Physical Activity Before, During, and After Chemotherapy for High-Risk Breast Cancer: Relationships with Survival. J. Natl. Cancer Inst..

[24] Kawaguchi K., Sakurai M., Yamamoto Y., Suzuki E., Tsuda M., Kataoka T.R., Hirata M., Nishie M., Nojiri T., Kumazoe M. (2019). Alteration of specific cytokine expression patterns in patients with breast cancer. Sci. Rep..

[25] Chen K., Lu P., Beeraka N.M., Sukocheva O.A., Madhunapantula S.V., Liu J., Sinelnikov M.Y., Nikolenko V.N., Bulygin K.V., Mikhaleva L.M. (2020). Mitochondrial mutations and mitoepigenetics: Focus on regulation of oxidative stress-induced responses in breast cancers. Semin. Cancer Biol..

[26] Sung H., Ferlay J., Siegel R.L., Laversanne M., Soerjomataram I., Jemal A., Bray F. (2021). Global Cancer Statistics 2020: GLOBOCAN Estimates of Incidence and Mortality Worldwide for 36 Cancers in 185 Countries. CA Cancer J. Clin..

[27] Hou M.-F., Ou-Yang F., Li C.-L., Chen F.-M., Chuang C.-H., Kan J.-Y., Wu C.-C., Shih S.-L., Shiau J.-P., Kao L.-C. (2021). Comprehensive profiles and diagnostic value of menopausal-specific gut microbiota in premenopausal breast cancer. Exp. Mol. Med..

[28] Byrd D.A., Vogtmann E., Wu Z., Han Y., Wan Y., Clegg-Lamptey J.N., Yarney J., Wiafe-Addai B., Wiafe S., Awuah B. (2021). Associations of fecal microbial profiles with breast cancer and nonmalignant breast disease in the Ghana Breast Health Study. Int. J. Cancer.

[29] Goedert J.J., Jones G., Hua X., Xu X., Yu G., Flores R., Falk R.T., Gail M.H., Shi J., Ravel J. (2015). Investigation of the association between the fecal microbiota and breast cancer in postmenopausal women: A population-based case-control pilot study. J. Natl. Cancer Inst..

[30] Stringer A.M., Gibson R.J., Bowen J.M., Keefe D.M. (2009). Chemotherapy-induced modifications to gastrointestinal microflora: Evidence and implications of change. Curr. Drug Metab..

[31] Montassier E., Gastinne T., Vangay P., Al-Ghalith G.A., Bruley des Varannes S., Massart S., Moreau P., Potel G., de La Cochetière M.F., Batard E. (2015). Chemotherapy-driven dysbiosis in the intestinal microbiome. Aliment. Pharmacol. Ther..

[32] Loman B.R., Jordan K.R., Haynes B., Bailey M.T., Pyter L.M. (2019). Chemotherapy-induced neuroinflammation is associated with disrupted colonic and bacterial homeostasis in female mice. Sci. Rep..

[33] Le Bastard Q., Ward T., Sidiropoulos D., Hillmann B.M., Chun C.L., Sadowsky M.J., Knights D., Montassier E. (2018). Fecal microbiota transplantation reverses antibiotic and chemotherapy-induced gut dysbiosis in mice. Sci. Rep..

[34] Daillère R., Vétizou M., Waldschmitt N., Yamazaki T., Isnard C., Poirier-Colame V., Duong C.P.M., Flament C., Lepage P., Roberti M.P. (2016). Enterococcus hirae and Barnesiella intestinihominis Facilitate Cyclophosphamide-Induced Therapeutic Immunomodulatory Effects. Immunity.

[35] Heshiki Y., Vazquez-Uribe R., Li J., Ni Y., Quainoo S., Imamovic L., Sørensen M., Chow B.K.C., Weiss G.J., Xu A. (2020). Predictable modulation of cancer treatment outcomes by the gut microbiota. Microbiome.

[36] Turnbaugh P.J., Ley R.E., Mahowald M.A., Magrini V., Mardis E.R., Gordon J.I. (2006). An obesity-associated gut microbiome with increased capacity for energy harvest. Nature.

[37] Vrieze A., Van Nood E., Holleman F., Salojärvi J., Kootte R.S., Bartelsman J.F.W.M., Dallinga-Thie G.M., Ackermans M.T., Serlie M.J., Oozeer R. (2012). Transfer of intestinal microbiota from lean donors increases insulin sensitivity in individuals with metabolic syndrome. Gastroenterology.

[38] Bajic J.E., Johnston I.N., Howarth G.S., Hutchinson M.R. (2018). From the Bottom-Up: Chemotherapy and Gut-Brain Axis Dysregulation. Front. Behav. Neurosci..

[39] Barandouzi Z.A., Starkweather A.R., Henderson W.A., Gyamfi A., Cong X.S. (2020). Altered Composition of Gut Microbiota in Depression: A Systematic Review. Front. Psychiatry.

[40] Paulsen J.A., Ptacek T.S., Carter S.J., Liu N., Kumar R., Hyndman L.K., Lefkowitz E.J., Morrow C.D., Rogers L.Q. (2017). Gut microbiota composition associated with alterations in cardiorespiratory fitness and psychosocial outcomes among breast cancer survivors. Support. Care Cancer.

[41] Deleemans J.M., Chleilat F., Reimer R.A., Baydoun M., Piedalue K.-A., Lowry D.E., Henning J.-W., Carlson L.E. (2022). The Chemo-Gut Pilot Study: Associations between Gut Microbiota, Gastrointestinal Symptoms, and Psychosocial Health Outcomes in a Cross-Sectional Sample of Young Adult Cancer Survivors. Curr. Oncol..

[42] Okubo R., Kinoshita T., Katsumata N., Uezono Y., Xiao J., Matsuoka Y.J. (2020). Impact of chemotherapy on the association between fear of cancer recurrence and the gut microbiota in breast cancer survivors. Brain Behav. Immun..

[43] Miller K.D., Nogueira L., Mariotto A.B., Rowland J.H., Yabroff K.R., Alfano C.M., Jemal A., Kramer J.L., Siegel R.L. (2019). Cancer treatment and survivorship statistics, 2019. CA Cancer J. Clin..

[44] Mcneely M.L., Sellar C., Williamson T., Shea-Budgell M., Joy A.A., Lau H.Y., Easaw J.C., Murtha A.D., Vallance J., Courneya K. (2019). Community-based exercise for health promotion and secondary cancer prevention in Canada: Protocol for a hybrid effectiveness-implementation study. BMJ Open.

[45] Carter S.J., Hunter G.R., Blackston J.W., Liu N., Lefkowitz E.J., Van Der Pol W.J., Morrow C.D., Paulsen J.A., Rogers L.Q. (2019). Gut microbiota diversity is associated with cardiorespiratory fitness in post-primary treatment breast cancer survivors. Exp. Physiol..

[46] Rashidi A., Kaiser T., Shields-cutler R., Graiziger C., Holtan S.G., Rehman T.U., Wasko J., Weisdorf D.J., Dunny G., Khoruts A. (2019). Dysbiosis patterns during re- induction/salvage versus induction chemotherapy for acute leukemia. Sci. Rep..

[47] Montassier E., Batard E., Massart S., Gastinne T., Carton T., Caillon J., Fresne S.L., Caroff N., Hardouin J.B., Moreau P. (2014). 16S rRNA Gene Pyrosequencing Reveals Shift in Patient Faecal Microbiota During High-Dose Chemotherapy as Conditioning Regimen for Bone Marrow Transplantation. Microb. Ecol..

[48] Godin G., Shephard R.J. (1985). A simple method to assess exercise behavior in the community. Can. J. Appl. Sport Sci..

[49] Amireault S., Godin G. (2015). The Godin-Shephard Leisure-Time Physical Activity Questionnaire: Validity Evidence Supporting its Use for Classifying Healthy Adults into Active and Insufficiently Active Categories. Percept. Mot. Ski..

[50] Cella D.F., Tulsky D.S., Gray G., Sarafian B., Linn E., Bonomi A., Silberman M., Yellen S.B., Winicour P., Brannon J. (1993). The Functional Assessment of Cancer Therapy scale: Development and validation of the general measure. J. Clin. Oncol..

[51] Lambert J.E., Parnell J.A., Han J., Sturzenegger T., Paul H.A., Vogel H.J., Reimer R.A. (2014). Evaluation of yellow pea fibre supplementation on weight loss and the gut microbiota: A randomized controlled trial. BMC Gastroenterol..

[52] Nicolucci A.C., Hume M.P., Martínez I., Mayengbam S., Walter J., Reimer R.A. (2017). Prebiotics Reduce Body Fat and Alter Intestinal Microbiota in Children Who Are Overweight or with Obesity. Gastroenterology.

[53] Bomhof M.R., Paul H.A., Geuking M.B., Eller L.K., Reimer R.A. (2016). Improvement in adiposity with oligofructose is modified by antibiotics in obese rats. FASEB J..

[54] Bolyen E., Rideout J.R., Dillon M.R., Bokulich N.A., Abnet C.C., Al-Ghalith G.A., Alexander H., Alm E.J., Arumugam M., Asnicar F. (2019). Reproducible, interactive, scalable and extensible microbiome data science using QIIME 2. Nat. Biotechnol..

[55] Sugiura K., Stock C.C. (1952). Studies in a tumor spectrum.I. Comparison of the action of methylbis(2-chloroethyl)amine and 3-bis(2-chloroethyl)aminomethyl-4-methoxymethyl-5-hydroxy-6-methylpyridine on the growth of a variety of mouse and rat tumors. Cancer.

[56] Abu Samaan T.M., Samec M., Liskova A., Kubatka P., Büsselberg D. (2019). Paclitaxel’s Mechanistic and Clinical Effects on Breast Cancer. Biomolecules.

[57] Cividalli A., Arcangeli G., Cruciani G., Livdi E., Cordelli E., Danesi D.T. (1998). Enhancement of radiation response by paclitaxel in mice according to different treatment schedules. Int. J. Radiat. Oncol. Biol. Phys..

[58] Seidman A.D., Berry D., Cirrincione C., Harris L., Muss H., Marcom P.K., Gipson G., Burstein H., Lake D., Shapiro C.L. (2008). Randomized phase III trial of weekly compared with every-3-weeks paclitaxel for metastatic breast cancer, with trastuzumab for all HER-2 overexpressors and random assignment to trastuzumab or not in HER-2 nonoverexpressors: Final results of Cancer and Leukemia Group B protocol 9840. J. Clin. Oncol..

[59] Reagan-Shaw S., Nihal M., Ahmad N. (2008). Dose translation from animal to human studies revisited. FASEB J..

[60] Van Der Meulen R., Makras L., Verbrugghe K., Adriany T., De Vuyst L. (2006). In Vitro Kinetic Analysis of Oligofructose Consumption by Bacteroides and Bifidobacterium spp. Indicates Different Degradation Mechanisms. Appl. Environ. Microbiol..

[61] Bachmanov A.A., Reed D.R., Beauchamp G.K., Tordoff M.G. (2002). Food intake, water intake, and drinking spout side preference of 28 mouse strains. Behav. Genet..

[62] Euhus D.M., Hudd C., LaRegina M.C., Johnson F.E. (1986). Tumor measurement in the nude mouse. J. Surg. Oncol..

[63] Parnell J.A., Reimer R.A. (2008). Differential Secretion of Satiety Hormones with Progression of Obesity in JCR:LA-corpulent Rats. Obesity.

[64] Silver N., Best S., Jiang J., Thein S.L. (2006). Selection of housekeeping genes for gene expression studies in human reticulocytes using real-time PCR. BMC Mol. Biol..

[65] O’Callaghan A., van Sinderen D. (2016). Bifidobacteria and Their Role as Members of the Human Gut Microbiota. Front. Microbiol..

[66] Ferreira-Halder C.V., Faria A.V.S., Andrade S.S. (2017). Action and function of Faecalibacterium prausnitzii in health and disease. Best Pract. Res. Clin. Gastroenterol..

[67] Pryde S.E., Duncan S.H., Hold G.L., Stewart C.S., Flint H.J. (2002). The microbiology of butyrate formation in the human colon. FEMS Microbiol. Lett..

[68] Tamanai-Shacoori Z., Smida I., Bousarghin L., Loreal O., Meuric V., Fong S.B., Bonnaure-Mallet M., Jolivet-Gougeon A. (2017). Roseburia spp.: A marker of health?. Future Microbiol..

[69] Baldelli V., Scaldaferri F., Putignani L., Del Chierico F. (2021). The Role of Enterobacteriaceae in Gut Microbiota Dysbiosis in Inflammatory Bowel Diseases. Microorganisms.

[70] Lee I.-A., Kim D.-H. (2011). Klebsiella pneumoniaeincreases the risk of inflammation and colitis in a murine model of intestinal bowel disease. Scand. J. Gastroenterol..

[71] Mirsepasi-Lauridsen H.C., Vallance B.A., Krogfelt K.A., Petersen A.M. (2019). Escherichia coli Pathobionts Associated with Inflammatory Bowel Disease. Clin. Microbiol. Rev..

[72] Coussens L.M., Werb Z. (2002). Inflammation and cancer. Nature.

[73] Hakansson A., Molin G. (2011). Gut Microbiota and Inflammation. Nutrients.

[74] Muscogiuri G., Cantone E., Cassarano S., Tuccinardi D., Barrea L., Savastano S. (2019). Gut microbiota: A new path to treat obesity. Int. J. Obes. Suppl..

[75] Bengmark S. (2013). Gut microbiota, immune development and function. Pharmacol. Res..

[76] Ortiz-Alvarez L., Xu H., Martinez-Tellez B. (2020). Influence of Exercise on the Human Gut Microbiota of Healthy Adults: A Systematic Review. Clin. Transl. Gastroenterol..

[77] Allen J.M., Mailing L.J., Niemiro G.M., Moore R., Cook M.D., White B.A., Holscher H.D., Woods J.A. (2018). Exercise Alters Gut Microbiota Composition and Function in Lean and Obese Humans. Med. Sci. Sports Exerc..

[78] Munukka E., Ahtiainen J.P., Puigbó P., Jalkanen S., Pahkala K., Keskitalo A., Kujala U.M., Pietilä S., Hollmén M., Elo L. (2018). Six-week endurance exercise alters gut metagenome that is not reflected in systemic metabolism in over-weight women. Front. Microbiol..

[79] Wan Y., Yuan J., Li J., Li H., Yin K., Wang F., Li D. (2020). Overweight and underweight status are linked to specific gut microbiota and intestinal tricarboxylic acid cycle intermediates. Clin. Nutr..

[80] Rothschild D., Weissbrod O., Barkan E., Kurilshikov A., Korem T., Zeevi D., Costea P.I., Godneva A., Kalka I.N., Bar N. (2018). Environment dominates over host genetics in shaping human gut microbiota. Nature.

[81] Campbell S.C., Wisniewski P.J., Noji M., McGuinness L.R., Häggblom M.M., Lightfoot S.A., Joseph L.B., Kerkhof L.J. (2016). The Effect of Diet and Exercise on Intestinal Integrity and Microbial Diversity in Mice. PLoS ONE.

[82] Durk R.P., Castillo E., Márquez-Magaña L., Grosicki G.J., Bolter N.D., Matthew Lee C., Bagley J.R. (2019). Gut microbiota composition is related to cardiorespiratory fitness in healthy young adults. Int. J. Sport Nutr. Exerc. Metab..

[83] Bressa C., Bailén-Andrino M., Pérez-Santiago J., González-Soltero R., Pérez M., Montalvo-Lominchar M.G., Maté-Muñoz J.L., Domínguez R., Moreno D., Larrosa M. (2017). Differences in gut microbiota profile between women with active lifestyle and sedentary women. PLoS ONE.

[84] Garrett W.S., Gallini C.A., Yatsunenko T., Michaud M., Dubois A., Delaney M.L., Punit S., Karlsson M., Bry L., Glickman J.N. (2010). Enterobacteriaceae Act in Concert with the Gut Microbiota to Induce Spontaneous and Maternally Transmitted Colitis. Cell Host Microbe.

[85] Pope J.L., Yang Y., Newsome R.C., Sun W., Sun X., Ukhanova M., Neu J., Issa J.-P., Mai V., Jobin C. (2019). Microbial Colonization Coordinates the Pathogenesis of a Klebsiella pneumoniae Infant Isolate. Sci. Rep..

[86] Mckee A.M., Kirkup B.M., Madgwick M., Fowler W.J., Price C.A., Dreger S.A., Ansorge R., Makin K.A., Caim S., Le Gall G. (2021). Antibiotic-induced disturbances of the gut microbiota result in accelerated breast tumor growth. iScience.

[87] Markou P., Apidianakis Y. (2014). Pathogenesis of intestinal Pseudomonas aeruginosa infection in patients with cancer. Front. Cell Infect Microbiol.

[88] Wennerberg E., Lhuillier C., Rybstein M.D., Dannenberg K., Rudqvist N.-P., Koelwyn G.J., Jones L.W., Demaria S. (2020). Exercise reduces immune suppression and breast cancer progression in a preclinical model. Oncotarget.

[89] Kim M.K., Kim Y., Park S., Kim E., Kim Y., Kim Y., Kim J.-H. (2020). Effects of Steady Low-Intensity Exercise on High-Fat Diet Stimulated Breast Cancer Progression Via the Alteration of Macrophage Polarization. Integr. Cancer Ther..

[90] Walsh N.P., Gleeson M., Shephard R.J., Woods J.A., Bishop N.C., Fleshner M., Green C., Pedersen B.K., Hoffman-Goetz L., Rogers C.J. (2011). Position statement. Part one: Immune function and exercise. Exerc. Immunol. Rev..

[91] Taper H.S., Roberfroid M. (1999). Influence of inulin and oligofructose on breast cancer and tumor growth. J. Nutr..

[92] Taper H.S., Roberfroid M.B. (2002). Inulin/oligofructose and anticancer therapy. Br. J. Nutr..

[93] Tandon D., Haque M.M., Gote M., Jain M., Bhaduri A., Dubey A.K., Mande S.S. (2019). A prospective randomized, double-blind, placebo-controlled, dose-response relationship study to investigate efficacy of fructo-oligosaccharides (FOS) on human gut microflora. Sci. Rep..

[94] Reimer R.A., Willis H.J., Tunnicliffe J.M., Park H., Madsen K.L., Soto-Vaca A. (2017). Inulin-type fructans and whey protein both modulate appetite but only fructans alter gut microbiota in adults with overweight/obesity: A randomized controlled trial. Mol. Nutr. Food Res..

[95] Su J., Li D., Chen Q., Li M., Su L., Luo T., Liang D., Lai G., Shuai O., Jiao C. (2018). Anti-breast Cancer Enhancement of a Polysaccharide from Spore of Ganoderma lucidum with Paclitaxel: Suppression on Tumor Metabolism with Gut Microbiota Reshaping. Front. Microbiol..

[96] Terrisse S., Derosa L., Iebba V., Ghiringhelli F., Vaz-Luis I., Kroemer G., Fidelle M., Christodoulidis S., Segata N., Thomas A.M. (2021). Intestinal microbiota influences clinical outcome and side effects of early breast cancer treatment. Cell Death Differ..

[97] Ramakrishna C., Corleto J., Ruegger P.M., Logan G.D., Peacock B.B., Mendonca S., Yamaki S., Adamson T., Ermel R., McKemy D. (2019). Dominant Role of the Gut Microbiota in Chemotherapy Induced Neuropathic Pain. Sci. Rep..

[98] Engels C., Ruscheweyh H.J., Beerenwinkel N., Lacroix C., Schwab C. (2016). The Common Gut Microbe Eubacterium hallii also Contributes to Intestinal Propionate Formation. Front. Microbiol..

[99] Kleessen B., Sykura B., Zunft H.J., Blaut M. (1997). Effects of inulin and lactose on fecal microflora, microbial activity, and bowel habit in elderly constipated persons. Am. J. Clin. Nutr..

[100] Frei R., Akdis M., O’Mahony L. (2015). Prebiotics, probiotics, synbiotics, and the immune system. Curr. Opin. Gastroenterol..

[101] Di Modica M., Gargari G., Regondi V., Bonizzi A., Arioli S., Belmonte B., De Cecco L., Fasano E., Bianchi F., Bertolotti A. (2021). Gut Microbiota Condition the Therapeutic Efficacy of Trastuzumab in HER2-Positive Breast Cancer. Cancer Res..

[102] Ahmed O.I., Adel A.M., Diab D.R., Gobran N.S. (2006). Prognostic value of serum level of interleukin-6 and interleukin-8 in metastatic breast cancer patients. Egypt. J. Immunol..

[103] Yoshimura T. (2018). The chemokine MCP-1 (CCL2) in the host interaction with cancer: A foe or ally?. Cell. Mol. Immunol..

[104] Mehta A.K., Kadel S., Townsend M.G., Oliwa M., Guerriero J.L. (2021). Macrophage Biology and Mechanisms of Immune Suppression in Breast Cancer. Front. Immunol..

[105] Barr M., Bouchier-Hayes D., Harmey J. (2008). Vascular endothelial growth factor is an autocrine survival factor for breast tumour cells under hypoxia. Int. J. Oncol..

[106] Mercurio A.M., Lipscomb E.A., Bachelder R.E. (2005). Non-angiogenic functions of VEGF in breast cancer. J. Mammary Gland. Biol. Neoplasia.

[107] Liang Y., Brekken R.A., Hyder S.M. (2006). Vascular endothelial growth factor induces proliferation of breast cancer cells and inhibits the anti-proliferative activity of anti-hormones. Endocr. Relat. Cancer.

[108] Wu X., Sun A., Yu W., Hong C., Liu Z. (2020). CXCL10 mediates breast cancer tamoxifen resistance and promotes estrogen-dependent and independent proliferation. Mol. Cell. Endocrinol..

[109] Kim M., Choi H.Y., Woo J.W., Chung Y.R., Park S.Y. (2021). Role of CXCL10 in the progression of in situ to invasive carcinoma of the breast. Sci. Rep..

